# TOFU-MAaPO: fast, scalable and reproducible analysis of large metagenome sequence data from the Sequence Read Archive

**DOI:** 10.1038/s41467-026-74033-9

**Published:** 2026-06-11

**Authors:** Eike Matthias Wacker, Malte Christoph Rühlemann, Andre Franke, David Ellinghaus

**Affiliations:** 1 Institute of Clinical Molecular Biology, Kiel University, Kiel, Germany; 2 Institute for Medical Microbiology and Hospital Epidemiology, Hannover Medical School, Hannover, Germany

**Keywords:** Clinical microbiology, Computational platforms and environments, Genetics research

## Abstract

Metagenomic shotgun sequencing data from over 600,000 metagenomes are publicly available in repositories such as NCBI’s Sequence Read Archive (SRA). Technically advanced and easy-to-use best-practice metagenome software workflows for raw data pre-processing, assembly of metagenome-assembled genomes, and taxonomic and functional annotation of metagenome-assembled genomes are needed for reproducible analysis and harmonization of large-scale metagenomic datasets. We introduce TOFU-MAaPO (Taxonomic Or FUnctional Metagenomic Assembly and PrOfiling), a portable, automated single-command Nextflow pipeline for large-scale analysis of metagenomic short-read sequencing data. It analyzes metagenome files locally or directly from the SRA using accession or study IDs. In a benchmark against three established metagenome software pipelines, the TOFU-MAaPO workflow yielded 12%, 42% to 77% more high-quality metagenome-assembled genomes, likely reflecting the integration of multiple complementary binning tools with a unified refinement strategy. Using its assembly-free taxonomic abundance profiling module, we also automatically downloaded 16,462 uniquely identifiable and accessible human gut metagenome samples from the SRA and taxonomically annotated them against the Genome Taxonomy Database on a high-performance cluster in less than 55 hours, including download time. TOFU-MAaPO makes large metagenome projects more accessible to individual research groups and is freely available at https://github.com/ikmb/TOFU-MAaPO.

## Abstract (web summary)

The authors introduce a portable, automated single-command Nextflow pipeline for large scale analysis of metagenomic short-read sequencing data. It makes large metagenome projects more accessible to individual research groups and is freely available.

## Introduction

Metagenome sequencing methods enable the analysis of the collective genomes of microbial communities in biological samples. However, comprehensive and high-quality reference genome catalogs are required for robust functional characterization and taxonomic classification of microbiome data. To catalog the human gut microbiome, for example, the Unified Human Gastrointestinal Genome (UHGG) project^1^ compiled more than 200,000 published reference genomes representing 4644 gut prokaryotes from multiple databases, without re-assembling from the raw data. Such collections support the detection and quantification of microorganisms in biological samples and are excellent for rough estimation of the microbial content of new sequenced samples, but naturally do not cover all ecological niches or host- and disease-associated microbal variation. To address this, we and others have generated species-level genome resources from specific patient cohorts. For example, we previously assembled 27,745 metagenome-assembled genomes (MAGs) from raw FASTQ files of 839 gut metagenomes from a prospective inflammatory bowel disease family cohort^2^, resulting in 1652 non-redundant species-level genome bins. Such catalogs provide an important basis for downstream analyses, including phenotype-specific microbiome-wide association studies (MWAS)^3^. Very large genome catalogs for different body regions are expected to be published in the near future, e.g., genome catalogs for different regions of the human body (The Million Microbiome of Humans Project^4^), which will further refine phenotype-specific studies. At the same time, the generation of such resources depends critically on robust, scalable, and reproducible computational workflows for processing and harmonizing large metagenomic datasets.

In general, phenotype-specific genome catalog studies, MWAS, and other metagenome analyses place high demands on the processing and harmonization of raw sequencing data. Differences in software selection, parameterization, and computational environments can substantially reduce reproducibility. From a software perspective, integrated workflow systems such as Nextflow^5^ or Snakemake^6^ are therefore strongly recommended^7^. Nextflow and Snakemake improve scalability, portability, reproducibility and ease of use by supporting modular workflow design, code sharing via platforms such as GitHub, BitBucket and GitLab, automated error handling, resumption of interrupted runs, visualization of software dependencies and workflow structure, use of software containers such as Docker^7^ and Apptainer (formely Singularity)^8^, and automated job control on high-performance computing (HPC) systems. From a user’s perspective, a sophisticated bioinformatics workflow for metagenomic analysis must provide quality control (QC), assembly of metagenomes, binning of contigs into species, estimation of pathway and taxonomic abundances either assembly-free or based on MAGs with subsequent bin refinement^9^ in a simple manner. Analytical pipelines^10–12^ and software packages^13–15^ for subtasks have been published for basic QC and processing of metagenome samples, but none of these pipelines combine all the steps required above. For example, MetaWRAP^12^ supports QC, taxonomic annotation, assembly, binning, and bin refinement. Yet, it does not feature pathway annotation. In addition, its software implementation does not integrate workflow management systems such as Nextflow or Snakemake, with corresponding limitations for parallelization and error handling. Similarly, the recently published pipeline metaFun^16^, although implemented in Nextflow, required multiple commands for different analysis steps and does not provide the same streamlined support for large-scale distributed execution on HPC systems. ATLAS^11^ provides QC, single-sample assembly, and bin refinement via Snakemake (Table 1) and is fast, but lacks the use of software containers and the ability to perform co-assembly and assembly-free taxonomic and pathway annotations. nf-core/mag^10^ is a Nextflow-based pipeline for metagenome assembly and binning that covers many of the required steps (so far without assembly free pathway annotation; Table 1). But even on an HPC, nf-core/mag results in long runtimes of several hours for processing a single metagenome, making the use of nf-core/mag unsuitable for processing and assembling hundreds, thousands, or even tens of thousands of raw metagenome samples from the SRA and creating genome catalogs. The microbiome community, therefore, lacks an easy-to-use (i.e., single-command) and efficient software tool for large-scale metagenome analysis implemented in a workflow manager environment that can accurately process hundreds or thousands of metagenomes in a complete pipeline in a short time.

**Table 1:** Comparison of TOFU-MAaPO’s functionality with metagenome software tools implemented with workflow management systems and used for quality and quantity benchmarks (Fig. 1)

	Features	TOFU-MAaPO	ATLAS	nf-core/mag	metaFun
Usability					
	Workflow management system	Nextflow	Snakemake	Nextflow	Nextflow
	Software container (Apptainer/Docker)	yes	no	yes	yes
	SRA Download	yes	no^a^	no	no
QC (Quality Control)					
	Reads trimming	yes	yes	yes	yes
	Contamination	yes	yes	yes	yes
	QC-Report	yes	yes	yes	yes
Assembly-free classification/abundance estimation					
	Taxonomical	yes	no	yes	yes
	Pathway	yes	no	no	yes
Assembly					
	Single-samples assembly	yes	yes	yes	yes
	Co-assembly possible	yes	no	yes	no
	Co-assembly by groups	yes	no	yes	no
Binning					
	Co-binning (one catalogue to bin to across all samples)	yes^b^	yes	yes	no
	Multiple binning software (number of binning tools)	yes (6)	yes (2)^c^	yes (3)	yes (2)
	Bin refinement	yes	yes^c^	yes	yes
	Refinement tool	MAGScoT	DAS Tool	DAS Tool	DAS Tool
Post processing					
	MAG quality check	yes	yes	yes	yes
	MAG abundance estimation	yes	yes	yes	no

^a^While ATLAS seemed to offer a similar automatic download functionality, which is described in the documentation, this feature is not available in recent versions of the pipeline.

^b^Co-binning in TOFU-MAaPO is being performed with the binning tool Vamb.

^c^ATLAS can either use two binning software tools together for refinement or use two others separately without refinement. A combination of all implemented binning tools is not possible.

**Fig. 1:**
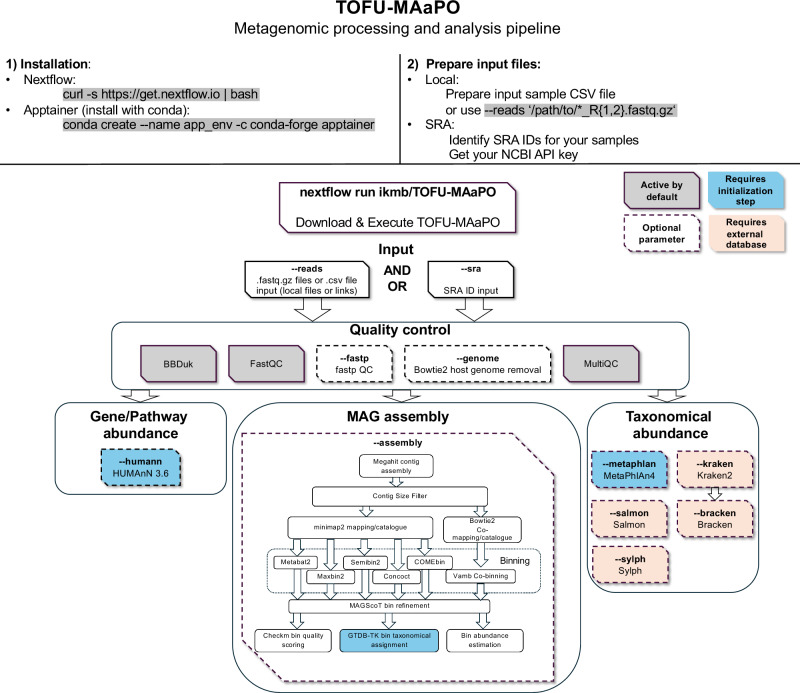
TOFU-MAaPO is a fast, easy-to-use, accurate, and efficient metagenomic software pipeline that processes metagenomic data available locally or from NCBI’s Sequence Read Archive (SRA). To run TOFU-MAaPO, the user simply needs to install Nextflow and Apptainer with two commands and then either enter a path to locally available FASTQ files or specify the SRA project (or its run or sample identifiers) for an automatic download from SRA. TOFU-MAaPO performs QC with trimming and artifact removal with the tools BBDuk and/or fastp, can optionally remove host reads with Bowtie2, and creates individual quality reports with FastQC and comprehensive MultiQC reports. After QC, the metagenomes can be analyzed in three different ways: pathway and gene abundance can be estimated with the assembly-free tool HUMAnN3. Taxonomic abundance can be estimated using the four different assembly-free tools MetaPhlAn4, Kraken2/Bracken, and Salmon, and with a high-throughput abundance profiling module including Sylph (see Fig. 2). In a third module, the pipeline enables the generation of metagenomic assembled genomes (MAGs) with the assembly tool MEGAHIT. The resulting contigs are then be binned with a selection of up to six different binning tools, these results are then merged and refined with MAGScoT.

Here we present TOFU-MAaPO (Taxonomic Or FUnctional Metagenomic Assembly and PrOfiling). TOFU-MAaPO (https://github.com/ikmb/TOFU-MAaPO) is a processing pipeline for metagenomic sequencing short reads and comprises four different modules, namely, quality control, gene/pathway abundance, MAG assembly and taxonomical abundance (Fig. 1). Through software optimizations and the use of the Apptainer container software in combination with Nextflow, TOFU-MAaPO can fully process metagenome samples four times faster than nf-core/mag. With minimal configuration based on Java, Nextflow, and Apptainer (quick installation also possible via Conda), TOFU-MAaPO’s modules for QC, taxonomical abundance, gene/pathway abundance, and genome assembly can be launched with a single-command without the user having to manually install software environments or software packages (with the exception of downloading certain databases for specific tools). New practical features include direct download and processing of sequencing data published in NCBI’s SRA, e.g., to easily create new phenotype-specific genome catalogs from raw data (FASTQ files) of the user’s choice, instead of compiling pre-assembled genomes from different analysis pipelines of different databases. The metagenomic assembly module in TOFU-MAaPO also provides a unique and improved bin refinement procedure using MAGScoT^17^ from results of up to six different binning tools.

First, for quality benchmark purposes, we measured the performance (runtime and accuracy) of TOFU-MAaPO compared to metaFun, ATLAS, and nf-core/mag by performing automated pipeline analyses with standard parameters for QC, assembly, binning, bin refinement, and post-processing of 100 publicly available human gut metagenomic samples. First, using TOFU-MAaPO, we created MAGs from all 100 metagenome samples and compared their accuracy with the MAGs generated by metaFun, nf-core/mag, and ATLAS. Second, we used TOFU-MAaPO’s taxonomic and pathway estimation modules to reproduce results of a study published in SRA using gut metagenomes from 50 healthy individuals and 50 patients with Myalgic encephalomyelitis/chronic fatigue syndrome (ME/CFS)^18^. Third, to demonstrate the scalability and potential of direct (re-)analysis of metagenome samples via TOFU-MAaPO’s SRA download function, we downloaded over 16,000 publicly available metagenomic samples with a single command procedure and assessed their taxonomic composition. We made all analysis benchmark scripts available at https://github.com/ikmb/TOFUpaper for the reproducibility of our results.

## Results

### Benchmark settings for QC and MAG assembly

Our aim was to perform a fair comparison between TOFU-MAaPO and the bioinformatic metagenome pipelines nf-core/mag^10^, ATLAS^11^, and metaFun^16^, which use Nextflow and Snakemake (Table 1), respectively, and to measure the quantity and quality of MAGs generated as well as the computing time of each pipeline. All three software pipelines focus on QC and the generation of MAGs. Before the benchmark, the required dependencies of each pipeline were installed, and a configuration file was set up for each pipeline. Details on the configuration and which parameters were set exactly when running the pipelines can be found in the Supplementary Methods. Details on which tools are used for which purpose in the QC and MAG assembly module of TOFU-MAaPO can be found in the “Methods” section. Where metaFun, ATLAS, or nf-core/mag use different tools compared to TOFU-MAaPO, we briefly explain this in the following text. Downstream analyses were also kept to a minimum and default settings, so that only taxonomic annotation and quality assessment was performed for each refined bin that was generated. All analyses were performed on the same benchmark compute system with two 32-core CPUs and one terabyte of RAM (see “Methods”). All analysis benchmark scripts are available for the purpose of reproducing the results at https://github.com/ikmb/TOFUpaper.

For benchmarking, we used SRA project SRP102150, which has 100 SRA IDs. While TOFU-MAaPO can download and preprocess data from SRA itself (see “Methods”), metaFun, ATLAS and nf-core/mag required these IDs to be manually downloaded and staged. It was also necessary to generate a CSV file containing file path information for nf-core/mag. For metaFun, ATLAS, and nf-core/mag, we therefore manually searched for the same metagenome files and downloaded them from SRA before starting the benchmark, which gives these pipelines a small runtime advantage per se, as TOFU-MAaPO downloads the data on-the-fly and then processes them locally. metaFun requires specific naming conventions for input files (*{1,2}.{fastq,fq}{.gz,}) located in a single directory. For nf-core/mag, it was further required to manually create a sample CSV file containing the IDs and paths to the locally stored files. For ATLAS, the CSV file could be created automatically with the initialization step in the ATLAS pipeline, which we manually modified to perform sample-specific binning (instead of the default co-binning in ATLAS) for direct quality comparison with metaFun, nf-core/mag and TOFU-MAaPO. To perform sample-specific binning, which is required for a variety of study designs, such as comparing microbial strains between samples, we manually ensured that unique values were used in the (binning) group column for each sample to perform single-sample binning in the automatically generated CSV input file. Because the default co-binning setting in the previous test exceeded our 3Tb limit for disk space, we elected to use single binning for ATLAS.

For QC, including trimming in TOFU-MAaPO, we used FastQC and BBDuk. ATLAS was run with FastQC and BBDuk as implemented in its workflow, whereas metaFun and nf-core/mag use fastp for trimming. TOFU-MAaPO also supports fastp as an alternative option (see “Methods”). In a further step, possible adapters and sequencing artifacts are removed with BBMap in TOFU-MAaPO (same in ATLAS) or with AdapterRemoval (unpublished) in nf-core/mag. The removal of the host genome sequences (here human) was carried out in all four pipelines with Bowtie2; removal of Phi X reads with Bowtie2 (nf-core/mag) or BBDuk (ATLAS; TOFU-MAaPO). MetaFun does not offer a Phi X removal step. nf-core/mag automatically downloads the host genome of choice at each pipeline call if not previously downloaded and specified; for the benchmark, we first downloaded the reference genomes and then started the pipeline with the locally available databases. TOFU-MAaPO allows users to create a list of different host genomes in a configuration file, enabling quick switching of host genomes between two runs. A comprehensive MultiQC output (also in nf-core/mag and something similar in ATLAS) was generated from all inputs and allows to check the QC performed.

Both ATLAS and nf-core/mag offer several assembler tools, and we chose the default assembler tool from the pipelines (MEGAHIT in metaFun, nf-core/mag, and TOFU-MAaPO; Spades in ATLAS). For bin refinement, ATLAS uses the two binning tools Maxbin2 and Metabat2. nf-core/mag also uses these two binning tools and CONCOCT, while metaFun uses Metabat2 and SemiBin2. After binning, all three pipelines refined the bins with DAS Tool^19^. TOFU-MAaPO uses a total of six binning tools with their default settings (Maxbin2, Metabat2, CONCOCT, COMEBin, Semibin2, and Vamb) followed by bin-refinement with MAGScoT (see “Methods”). TOFU-MAaPO and nf-core/mag evaluate the quality of the refined bins with CheckM (see “Methods”). ATLAS and metaFun use CheckM2^20^ for the quality assessment of refined bins. All four pipelines perform taxonomic annotation with GTDB-Tk (see “Methods”).

### Benchmark results for QC and MAG assembly

All four pipelines performed the high-standard QC steps with removal of human host sequencing reads followed by metagenome assembly, binning, and bin refinement with their implemented tools for the benchmark metagenomic data from 100 faecal metagenomes. The ATLAS pipeline stopped due to an unrecoverable error caused by the lack of an intermediate file. We were able to restart and finish the interrupted benchmark run for ATLAS after splitting the input files into two parts and added both runtimes for a total runtime from start to finish. TOFU-MAaPO, metaFun, and nf-core/mag finished the benchmark runs without errors.

**Fig. 2:**
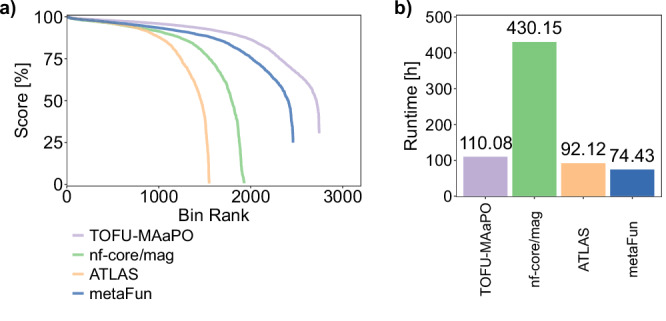
Results of the QC and MAG assembly benchmark for the dataset SRP102150 (containing 100 human gut metagenomic samples) downloaded from the SRA and processed with the pipelines TOFU-MAaPO, nf-core/mag, ATLAS, and metaFun, with settings set to single-sample-binning and bin refinement enabled. **a** Each metagenome assembly pipeline returned refined bins that were taxonomically annotated and assigned a quality score. Refined bins were scored for completeness and contamination in TOFU-MAaPO and nf-core/mag using CheckM (see “Methods”). For ATLAS and metaFun, CheckM was run separately for the pipeline outputs. A single score was calculated by Score = Completeness – 0.5*Contamination. **b** Runtime assessment of the four pipelines running sequentially on the same benchmark computer system (see “Methods”). For metaFun, ATLAS, and nf-core/mag, we had to manually download the same metagenome files from SRA before starting the benchmark, which gave all three pipelines a small runtime advantage per se, since TOFU-MAaPO downloads the files on-the-fly and then processes them locally.

We compared the quality and quantity of MAGs using the CheckM quality score, which was directly available from TOFU-MAaPO and nf-core/mag and was generated manually for the results from ATLAS and metaFun. CheckM (see “Methods”) is a widely adopted standard for assessing final MAG quality in metagenomic studies. We found that the numbers of MAGs and the quality of the MAGs in the four pipelines differed significantly (Fig. 2a). With 2745 high-medium MAGs in total (here CheckM score ≥ 0.306 due to a required completeness score ≥ 0.5 in MAGScoT; MAGScoT does not output MAGs with lower scores as these are generally discarded due to quality issues), TOFU-MAaPO generated significantly more MAGs than metaFun (2461 MAGs), nf-core/mag (1929 MAGs) and ATLAS (1548 MAGs) with their bin refinement procedures (no score filtering at all; including MAGs of very low quality). In our benchmark, the TOFU-MAaPO workflow yielded 12, 42, and 77% more high-quality bins (when filtering with CheckM score ≥0.5) than metaFun, nf-core/mag, and ATLAS, respectively, after their respective bin refinement procedures, likely reflecting the combined effect of integrating multiple complementary binners with a unified refinement strategy. Further, we observed remarkably different run times for the pipelines (Fig. 2b). While TOFU-MAaPO, metaFun and ATLAS assembled, binned, refined, scored, and annotated the bins for all 100 samples in less than four days (110.08 h for TOFU-MAaPO including data download time; 74.43 h for metaFun and 92.12 h for ATLAS excluding data download time), nf-core/mag took over 17 days (430.15 h excluding data download time) on the benchmark compute system. We identified the implementation of the CONCOCT binning tool in nf-core/mag (showing library warnings during use) as the primary factor for the exceptionally long runtime compared to ATLAS and metaFun, which do not feature CONCOCT and TOFU-MAaPO with implemented CONCOCT. Our analysis indicates that the unexpectedly longer total runtime in nf-core/mag is largely attributable to the way the CONCOCT binning step is implemented within that workflow, which introduces substantial overhead from numerous auxiliary processes such as compression and decompression tasks. By contrast, the CONCOCT module in TOFU-MAaPO showed substantially better performance, requiring on average 2.23 h with 8 cores per sample compared with 8.38 h with 12 cores per sample in nf-core/mag. This difference is consistent with a more efficient parallelization strategy in TOFU-MAaPO. ATLAS produced about half as many MAGs as TOFU-MAaPO (56%) while using four fewer binning tools and requiring a shorter runtime. metaFun showing the lowest run time could be explained by choosing two binning tools with moderate run times. Details on the replication of results from the original study^18^ using the public NCBI-SRA dataset SRP102150 with TOFU-MAaPO are provided in the Supplementary Note and Supplementary Fig. 1.

### SRA high-throughput taxonomic abundance profiling

**Fig. 3:**
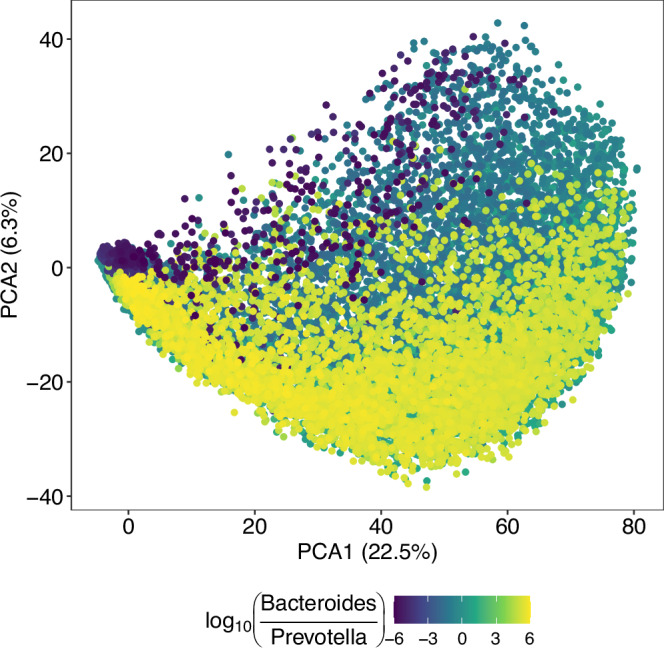
Results of Aitchison principal component analysis (PCA) for 16,462 uniquely identifiable and accessible human gut metagenome samples downloaded from the SRA and processed using the high-throughput taxonomic abundance module of TOFU-MAaPO with a single command. The reference database for estimation of taxonomic abundance contained all genomes from the Genome Taxonomy Database Release 220 (specified as the default database for Sylph in TOFU-MAaPO, with currently 113,104 bacterial and archaeal species representative genomes, see “Methods”). Taxonomic abundance was estimated on genus level. For visualization, results were merged in the R environment, centered log ratio (CLR) transformed, and colored by the logarithmic abundance of the genus *Bacteroides* divided by the abundance of *Prevotella*.

Assembly-based analyses with generation of MAGs are computationally intensive, as shown in the QC and assembly benchmark results (Fig. 2b), especially if more than a few hundred metagenomes are to be analyzed. High-throughput profiling methods (without assembly) are therefore a fast alternative for estimating of taxonomic abundance for thousands of metagenomes. We implemented a separate high-throughput taxonomical abundance module for fast and efficient (assembly-free) estimation of taxonomic abundance, which also works for low coverage genomes where assembly is not possible. TOFU-MAaPO’s high-throughput taxonomical abundance module can directly process thousands or tens of thousands of samples located in the SRA in combination with the abundance-corrected k-mer method implemented in Sylph (see “Methods”). The user only needs to provide a list of SRA project or SRA sample/run IDs (see “Methods”), their NCBI API key, and optionally a custom Sylph database to start the download process of gzipped FASTQ files from SRA and process them with Sylph. As an example of a single-command analysis with TOFU-MAaPO on our benchmark computer system, we downloaded, analyzed, and compared 16,462 uniquely identifiable and accessible human gut metagenome samples from the SRA listed in the ExperimentHub database^21^ (see “Methods”) with Genome Taxonomy Database (GTDB) release 220^22^ (specified as the default database for Sylph in TOFU-MAaPO), which currently includes 113,104 bacterial and archaeal species. The process was limited by the sample data download, as we downloaded only up to ten samples simultaneously. We performed the process in 18 batches with up to 1000 samples and measured a summarized runtime of 54 h and 13 min for the entire process, which temporarily required up to 4.3 terabytes of disk space per batch on the HPC. In total 39.2 terabytes of data were downloaded and/or written on the disk space for this analysis. The results of a subsequent Aitchison principal components analysis (PCA) at the genus level (see “Methods”) show that *Bacteroides* and *Prevotella* are main drivers of the taxonomical diversity of the gut microbiome in these samples (Fig. 3). The results show that TOFU-MAaPO quickly and accurately estimates taxonomic frequencies for rare taxa or metagenomic data with low coverage that are difficult to assemble, without the need to perform the computationally intensive assembly process described above.

## Discussion

We developed the TOFU-MAaPO software pipeline with the aim of providing scientists with an easy-to-use method to rapidly process large numbers of metagenomes in a scalable and reproducible way, while optimally utilizing the underlying computational capacities. It reduces the enormous effort for scientists to install numerous software tools and chain them together in large workflows to achieve state-of-the-art results. TOFU-MAaPO is executable via single commands without the need to manually install and curate software packages. Each process downloads and executes only the required software container, allowing users to focus entirely on analyzing their metagenomic data.

We show that TOFU-MAaPO can create high-quality MAGs by using, to our knowledge, a unique application of six binning tools. The results of all six binning tools are then combined and refined into a single set of high-quality bins, which are then taxonomically characterized. Integrating multiple complementary binning approaches can improve MAG recovery because different methods capture partly distinct subsets of genomes. Previous benchmarking and review studies have shown that combining and refining results across multiple binners can improve overall MAG reconstruction, although this benefit depends on the dataset and analysis strategy used^23,24^. The user can also deactivate binning tools if computational speed is of higher priority than the quality of results.

TOFU-MAaPO is a multi-purpose pipeline that not only performs QC and assembly of metagenomes, but also contains modules for non-assembly based estimation of taxonomic or gene abundance. Users can process large metagenomic data from NCBI’s SRA with a variety of tools implemented in a single pipeline. TOFU-MAaPO generates output tables and MAGs that serve as the basis for subsequent statistical and comparative analysis. The pipeline is well suited for processing large amounts of data in an extremely short time by an implementation of a high-throughput module for rapid assessment of taxonomic abundance in metagenomic data without assembly.

As the software continues to evolve, we will further develop the pipeline by adopting new software techniques and adding new tools. However, in future versions of TOFU-MAaPO, we will keep old tools available and make new tools available in a modular way. Thus, by default, the user is presented with a best practice workflow, but can also use older tools within the workflow to reproduce previous results. We are planning to add additional assembly tools and binning tools to TOFU-MAaPO. We also plan to provide additional modules and stand-alone pipelines covering different aspects of metagenomic data processing. This includes the dereplication of assembled MAGs on a cohort scale to provide the highest quality MAG candidates for user-selected percentages of average nucleotide identity.

We plan to integrate tools such as EggNOG^25,26^ into TOFU-MAaPO to facilitate the direct creation of gene-catalogues from downloaded metagenomic data and to broaden the scope of biological insights that can be gained from such data. This will make large metagenome and gene catalog projects increasingly feasible for individual research groups, including projects that previously often required the coordinated effort of large consortia such as the UHGG Project^1^. Software frameworks such as TOFU-MAaPO, based on technologies such as Nextflow and Apptainer, can therefore enable not only more efficient analysis but also broader access to large-scale metagenomic data science.

## Methods

### Nextflow implementation

TOFU-MAaPO was written in Domain-specific language 2 of the free and open-source Nextflow^5^ pipeline management framework to efficiently program and parallelize processes on various compute systems. These include desktop computers and HPC systems equipped with a job scheduler system such as Slurm, which ensures the best possible utilization of available computer resources to run as many jobs as possible simultaneously. The pipeline requires Nextflow (with Java 17 or higher) and Apptainer^8^, the community-maintained successor of Singularity. To easily install the dependencies via Conda^27^ and to run the four different modules via single commands, we provide a quick start guide at https://github.com/ikmb/TOFU-MAaPO/blob/master/README.md#quick-start.

The standard Nextflow command line call with FASTQ input data (details below) only executes the QC module (Fig. 3). By default, the pipeline is terminated after completion of the QC-module if no other module is activated. The gene/pathway abundance, MAG assembly, and taxonomical abundance modules are only executed if the user wants to perform a specific analysis. In this way, the user receives the desired results as quickly as possible, while unwanted output is reduced to a minimum. Especially with a multi-user computer system such as an HPC, the user’s computational footprint should be as low as possible. Nextflow workflows use automatic caching and can be resumed after interruption from the last completed step. This functionality also allows users to reuse results from previously completed runs when activating additional modules, without restarting the workflow from the beginning. For example, all processes of the QC module can be cached to directly execute the not yet performed metagenomic genome assembly module if in an earlier run only a reference-based taxonomic abundance estimation for the identical input samples was performed.

### Hardware requirements

For a complete metagenomic analysis workflow, including the MAG assembly module, we recommend at least 16 CPU cores and 128 GB RAM as well as at least 1 TB of available free hard disk space. The amount of free hard disk space is variable, as it largely depends on the number of metagenomes used, their sequencing depth, and the software tools selected. The pipeline creates a temporary working directory containing all temporary files. If the host system has a scratch directory, the pipeline can use this to reduce the storage size of the temporary working directory. At the end of the run, the temporary data can be removed. A dedicated example configuration file can be found on https://github.com/ikmb/TOFU-MAaPO/blob/master/docs/installation.md.

### Containerization, error handling, and versioning

Within the modules in TOFU-MAaPO, a software container is defined for each sub-process (currently 63 in number), which provides all implemented tools and is independent of the other sub-processes. This modularization ensures small container sizes with short container initialization times and facilitates maintenance and expansion with new process elements. We implemented and used Apptainer as default container software for this pipeline, as this software is intended for scientific usage with user rights and does not require higher privileges (superuser permissions of administrators) for execution. However, many of the sub-processes in TOFU-MAaPO are implemented as Docker containers (e.g., the MEGAHIT program) since Apptainer can read and execute the Docker standard. This has the advantage that the default container software (in this case, Apptainer) does not require superuser rights from administrators, and, for example, sub-process Docker containers can be executed without superuser rights. Other default container software solutions instead of Apptainer (e.g., Docker, Podman), can be used if they are configured in the respective configuration file (see https://www.nextflow.io/docs/latest/container.html). If a TOFU-MAaPO run is not completed successfully, the pipeline will restart the run with more computer resources up to a user-defined maximum (an advanced retry function) of computer resources and/or number of retries. Pipeline versioning allows the use of older versions of TOFU-MAaPO to be used with the corresponding containers to enable reproduction of results from older TOFU-MAaPO calls. The pipeline also reports a list of actively used software versions for each run. Details of the software tools currently in use can be found in the following sections and Supplementary Table 1.

### Databases

TOFU-MAaPO requires databases and auxiliary data for some tasks. We have implemented single-command initialization steps for the three modules gene/pathway abundance, MAG assembly, and taxonomical abundance (for details, see sections below). The corresponding databases can then be downloaded to user-defined system paths before the respective analysis tool is executed. This leads to the databases only having to be downloaded at the time they are needed. For tools that can run with different databases (Kraken2/Bracken, Salmon, Sylph), we provide recommendations for database sources that can be used with TOFU-MAaPO, see https://github.com/ikmb/TOFU-MAaPO. For these, the user must download the respective database and specify the path to the database location. To use a host genome for decontamination (removal of host reads), it is necessary that it is available as a Bowtie2 index. We provide a link to publicly available Bowtie2 genome indices at https://github.com/ikmb/TOFU-MAaPO, as well as a tutorial on how to create a new index from available genomes from NCBI (https://github.com/ikmb/TOFU-MAaPO/blob/docs/docs/hostgenome.md).

### Input files and SRA download function

The input for TOFU-MAaPO are either (i) gzipped FASTQ files available locally on hard disk, which can be specified directly by a command-line call (parameter –reads) via a three or four column csv file containing the sample IDs in one column and the path information to the sequence reads (two columns for paired-end sequencing, one column for single-end sequencing reads) in two or three columns, or (ii) SRA project or sample IDs from the SRA database (parameter –sra) (Table 1). A combination of both input methods (i and ii) is also possible. If SRA IDs were selected as input, the user must specify the personal NCBI API key (see https://ncbi.nlm.nih.gov/account/settings/) together with the SRA IDs as a comma-separated list. TOFU-MAaPO queries the IDs from NCBI’s SRA servers and returns a list of matching elements with the path to the associated FASTQ files (either paired, unpaired, or paired and unpaired FASTQ files). The pipeline then downloads the query results sequentially using the Linux curl command, allowing for up to ten downloads in parallel in the default configuration, and begins processing for each successfully downloaded sample. This approach allows the pipeline to efficiently utilize the free computing capacity, i.e., perform QC processes while other samples are still being downloaded.

### Quality control (QC) module

By default, TOFU-MAaPO performs QC for all input files. Prior to QC, the quality of the unprocessed metagenomic reads is assessed with FastQC^28^. Reads are trimmed and adapter sequences (Default: Illumina Nextera adapter sequences) are removed with BBDuk of the BBTools software suite^29^ and the following parameters: “ktrim = r k = 23 mink = 11 hdist = 1 minlength = 50 tpe tbo”. Sequencing artifacts and possible Phi X contaminations are removed with BBDuk with the parameters: “k = 31 ref = artifacts,phiX ordered cardinality minlength = 50”. We also offer the optional use of fastp via the flag “--fastp”^30^, a fast tool used for trimming and as an alternative to quality assessment by FastQC. The removal of host contamination reads is recommended but optional. For this, the user must provide a host reference genome in the Bowtie2 format. We provide links to a publicly available repository for frequently used genomes and a small tutorial how to create a Bowtie2 index from any genome available from NCBI (see section Databases above). If host genome read removal is enabled via “--genome”, all reads are aligned to the reference genome with Bowtie2^31^ and the parameters “–met-stderr –no-unal –sensitive –end-to-end”. Only unaligned reads are used further downstream. At the end of QC, a summarized MultiQC^32^ report is generated for all input samples. For locally available, already quality-controlled inputs, the pipeline provides a shortcut that skips the QC module.

### Gene/pathway abundance module

When activated via “--humann”, HUMAnN (v3.6) is used for functional profiling and pathway estimation and estimates the abundance of molecular functions from sequencing data without the need for prior translation into potential amino acid structures. HUMAnN is remarkable for its ability to stratify functions based on their microbe of origin^33^. HUMAnN requires a separate database to run successfully, and TOFU-MAaPO loads this automatically at the initialization step of this module via command “--updatehumann” (Further described at https://github.com/ikmb/TOFU-MAaPO/blob/master/docs/installation.md#metaphlan--humann), i.e., when the gene/pathway abundance module is used for the first time.

### MAG assembly module

When activated via “--assembly”, the MAGs assembly module uses the quality-controlled sequencing reads and performs contig assembly with MEGAHIT^34^, a tool optimized for memory-efficient assembly from metagenomic NGS data. If the input to TOFU-MAaPO is a CSV file, a column “group” together with the parameter “--assemblymode group” can be used to specify which samples should be co-assembled with MEGAHIT. For all other input options, the options for “--assemblymode” are either “single” (default setting), which assembles all samples individually, or “all”, which assembles all samples together. Contigs will be filtered to a desired minimum length (by default 2000bp). The filtered output will then be catalogued and indexed with minimap2^35^ and the corresponding sample reads are mapped individually to the contig catalogue. The resulting bam files are used to score the individual contig depths with the MetaBAT2’s utility script jgi_summarize_bam_contig_depths.

The generated files are the input files for the activated binning tools (process of read or contig grouping with assignment to individual genomes), which are freely selectable with all (default) or at least one from a list of six binning tools MetaBAT2^36^, COMEBin^37^, Concoct^38^, Maxbin2^39^, SemiBin2^40^, and VAMB^41^. We configured all binning tools, with the exception of VAMB, in such a way that all contigs belonging to a sample are binned together. For VAMB, a co-binning approach was configured for default use, as this is the recommended usage by the binning tool authors. In the default setting, the samples are divided into groups of up to 100 samples for co-binning. It is possible to change the group size to either reduce computational time of VAMB (high group numbers lead to higher computational time) or to allow co-binning of all input samples in a single group.

For post-processing, incomplete bins are refined with MAGScoT^17^ by combining them with other matching bins based on a profile of the presence of single-copy marker genes detected with prodigal^42^ and stored as HMM-profiles^43,44^) from the Genome Taxonomy Database Toolkit (GTDB-Tk^45,46^); in each bin. Also, all bins are scored with MAGScoT, so that only bins that received a higher score than a threshold (default 0.5) are used as refined output. These bins are then independently scored again for completeness and contamination with the community tool CheckM^47^, nowadays the *de facto* standard in almost every large-scale metagenome study, and taxonomically annotated with GTDB-Tk. To use GTDB-Tk, the respective GTDB database must be downloaded to the user’s computer system into a user-specified directory, which is done the first time the assembly module is used (“–updategtdbtk”, only for first-time initialization; https://github.com/ikmb/TOFU-MAaPO/blob/master/docs/installation.md#metaphlan–humann). Finally, a user-defined script is used to estimate the relative abundance of refined bins in each sample, and the resulting individual sample abundances are merged into an output table.

### Taxonomical abundance module

TOFU-MAaPO offers four different tools for estimating the taxonomical abundance per sample so that a tool can be selected according to the user’s individual preference for specific ecosystems: MetaPhlAn^48^, Kraken2^49^, with/without Bracken^50^, Sylph^51^, and Salmon^52^. These assembly-free estimation tools rely on a database that must cover the organisms present in the metagenomic sample to be analyzed. For MetaPhlAn, we provide an automated initialization step (–updatemetaphlan) that is executed when using the software tool for the first-time to download the required database curated by the authors of the tool (https://github.com/ikmb/TOFU-MAaPO/blob/master/docs/installation.md#metaphlan--humann). For Kraken2, we describe how to download a curated database (https://github.com/ikmb/TOFU-MAaPO/blob/master/docs/installation.md#kraken2).

MetaPhlAn is an alignment-based profiling tool based on unique clade-specific marker genes. With the release of version 4.0 of MetaPhlAn, the reference database has grown to about one million microbial genomes, which covers a broad range of potential metagenomic ecosystems. Kraken2 is a k-mer based sequence classifier that assigns taxonomical labels to individual DNA sequences. It improved from Kraken^53^, which assigns the lowest common ancestor taxa to each k-mer by using a probabilistic table of hashes to reduce memory requirements. Bracken can then be used to improve the results of Kraken2 to obtain more accurate estimates of taxonomic abundances at the species and genus level based on a Bayesian approach to redistribute reads along the taxonomic tree. For additional usage we also integrated Salmon, a tool originally developed specifically for RNA-sequencing data and can also be used to quantify metagenomic data quickly by activating a “meta-flag”. For Kraken2/Bracken, MetaPhlAn, and Salmon, TOFU-MAaPO merges the per sample results into a single combined output table. For the module Sylph, TOFU-MAaPO can either publish a separate output table for each sample (default) or a combined output table. For Sylph, we implemented its default database, which is based on a GTDB curated by the developers.

### Metagenomic data used for MAG assembly quality benchmarks and taxonomic and pathway estimation reproducibility analysis

We used a publicly available dataset of 100 faecal metagenomes from ME/CFS patients and controls stored in the NCBI Sequence Read Archive with ID SRP102150^18^ to compare the ease of use, computational time, and quality of results of the workflow managed metagenome pipelines nf-core/mag (v3.0.2-gbb74c46), ATLAS (v2.18.2), metaFun (v1.0.0), and TOFU-MAaPO (v1.6.0). The dataset was generated using an Illumina HiSeq 4000 and included 50 control samples and 50 patient samples. We further used this dataset for taxonomic and pathway estimation with TOFU-MAaPO. A system with Ubuntu 22.04.4 LTS and two 32-core CPUs (2× Intel(R) Xeon(R) Gold 6242 CPU @ 2.80 GHz) was used as benchmark computer. The system also contained 1 terabyte of RAM and 3 terabytes of SSD data storage. We measured runtimes with CMDBench^54^.

### Metagenomic data downloaded from SRA to determine the taxonomic composition

To demonstrate the ability of scalability and potential of direct re-analysis of metagenome samples via TOFU-MAaPO’s SRA download functionality, we downloaded over 17,090 publicly available metagenomic samples with TOFU-MAaPO and assessed their taxonomic composition (without assembly). The computation was performed on a HPC with one head node scheduling jobs with SLURM to 55 compute nodes and one dedicated data download and mover node with a user storage quota of 40 terabytes. The sample annotation was taken from the ExperimentHub database via the Bioconductor package curatedMetagenomicData version 3.8.0^21^ and uses SRA IDs. If multiple SRA run IDs belonged to the same sample SRA ID in ExperimentHub, only the first listed SRA run ID was used (in longitudinal studies, SRA run IDs of different time points are assigned to the same SRA sample IDs). In total, 17,934 SRA run IDs from this source were taken and 17,090 run IDs could be successfully automatically queried from the SRA via TOFU-MAaPO and their data downloaded. To save disk space, the run IDs were split into batches of up to 1000 samples, this allowed the removal of intermediate files after each batch processing. Runtime duration for each batch was extracted from the reports for each batch, which are generated by Nextflow and summed up to a total runtime. Taxonomic composition analysis with Sylph yielded non-empty results for 16,462 SRA run IDs. Data was further analyzed in R (v4.3.3) with the packages tidyverse^55^. Aitchison principal component analysis was performed by using the genus-level abundance data and adding a pseudocount to each value and performing centered log ratio transformation. The principal component analysis was calculated with the package irlba^56^, and the result was visualized with ggplot2^57^.

### Reporting summary

Further information on research design is available in the Nature Portfolio Reporting Summary linked to this article.

## Supplementary information

Supplementary Information

Reporting Summary

Transparent Peer Review file

## Data Availability

The sequencing data used in this study are available from the Sequence Read Archive (SRA) under accession SRP102150. Further SRA accession numbers are listed in the project repository https://github.com/ikmb/TOFUpaper.

## Code availability

TOFU-MAaPO is available on GitHub (https://github.com/ikmb/TOFU-MAaPO) and archived at Zenodo^58^. All used scripts for benchmarking and analysis can be found at https://github.com/ikmb/TOFUpaper.
