# Supplementary material for: TOFU-MAaPO: fast, scalable and reproducible analysis of large metagenome sequence data from the Sequence Read Archive
Source: Nat Commun. 2026 Jun 11;17:5215. doi: 10.1038/s41467-026-74033-9 (PMC13260335; doi:10.1038/s41467-026-74033-9)
Supplement: Supplementary file 1 — Supplementary Information [file 41467_2026_74033_MOESM1_ESM.pdf]

Supplementary Information

TOFU-MAaPO: Fast, scalable and reproducible analysis of large metagenome sequence data from the Sequence Read Archive

Eike Matthias Wacker, Malte Christoph Rühlemann, Andre Franke, David Ellinghaus

Supplementary Methods 2
- **Configuration file for nf-core/mag and TOFU-MAaPO** 2

Supplementary Notes 8
- **Performing a benchmark between TOFU-MAaPO, ATLAS, metaFun and nf-core/mag** 8
- **Replication of published results of the NCBI SRA public dataset SRP102150** 11

Supplementary Tables 12

Supplementary Figures 15

References 16

Supplementary Methods

Configuration file for nf-core/mag and TOFU-MAaPO

In the benchmarks between TOFU-MAaPO and nf-core/mag for MAG assembly on the same system both pipelines required a preset configuration. For TOFU-MAaPO, the configuration profile "biomedinf" was used (See: https://github.com/ikmb/TOFU-MAaPO/blob/master/conf/biomedinf.config). The file TOFU.config contained following entries:

```
params {
  //Software DB locations, UNCOMMENT AND CHANGE THEM:


  metaphlan_db = "/dpool/ewacker/metagenomics/TOFU-MAaPO/quickstart/databases/Metaphlan/4.0"
  //kraken2_db = "/dpool/ewacker/metagenomics/TOFU-MAaPO/quickstart/databases/Kraken2/k2_viral_20210517"
  humann_db = "/dpool/ewacker/metagenomics/TOFU-MAaPO/quickstart/databases/Humann3/3.6"
  gtdbtk_reference = "/dpool/ewacker/metagenomics/TOFU-MAaPO/quickstart/databases/GTDB-TK/release207_v2"


  //For host read removal list your host genomes as bowtie2 index in this named list with full path to the basename of the index:
'genomes' {
'human' {
```

```
    bowtie_index = "/dpool/ewacker/metagenomics/TOFU-MAaPO/quickstart/genomes/GRCh38_noalt_decoy_as/GRCh38_noalt_decoy_as"
}
}

  // MAXIMUM PER PROCESS CONFIGS, CHANGE THEM TO YOUR HARDWARE SPECS
  max_memory = 500.GB
  max_cpus = 64
  max_time = 100.h

  //Scratch. Does your system support scratch? If not, leave it false
  scratch = false
}

//Enable Singularity as container software
singularity {
enabled = true
  // Singularity configs, CHANGE THEM TO YOUR USED FILESYSTEM, if not properly set the container won't see your files
runOptions = "-B /home -B /dpool"
// where should the containers be downloaded to
cacheDir = "/dpool/ewacker/metagenomics/TOFU-MAaPO/quickstart/singularity_cache"
```

```
}

process {
  //Default executor for each process, other options can be e.g. SLURM.
  //See https://www.nextflow.io/docs/latest/executor.html for more options and details.
  executor='local'
}

//Default for total execution, remove this whole part if you are using a different option above than executor='local':
executor {
  cpus = 64
  memory = 900.GB
}
```

The custom configuration file for nf-core/mag [1] consisted out of following:

```
params {
  // Defaults only, expecting to be overwritten
  max_memory = 500.GB
  max_cpus = 64
  max_time = 100.h
```

```
  //igenomes_base = '/dpool/ewacker/metagenomics/metagenomic-workflows/databases/iGenomes/references/'
  saveReference = true

}

  //      'genomes' {
//         'human' {
      //     fasta = "/dpool/ewacker/metagenomics/metagenomic-workflows/databases/human/GRCh38_noalt_decoy_as/hs32d1_fasta/GCA_000001405.15_GRCh38_no_alt_plus_hs38d1_analysis_set.fna"
//         bowtie2 = "/dpool/ewacker/metagenomics/metagenomic-workflows/databases/human/GRCh38_noalt_decoy_as/hs32d1_bowtie2/"
//                    }
//    }

//Defaults for each process:
process {
    executor='local'

    withName: CONCOCT_CONCOCT {
```

```
    time   = 72.h
  }

  withName: GTDBTK_CLASSIFYWF {
        cpus = 32
        memory = 300.GB
  }
  withName: CHECKM_QC {
        cpus = 32
        memory = 300.GB
  }
  withName: CHECKM_LINEAGEWF {
        memory = 300.GB
        cpus = 1
        container = "docker://quay.io/biocontainers/checkm-genome:1.1.3--py_1"
  }
}

//Default for total execution, remove this part when not executor='local':
executor {
  cpus = 64
```

```
  memory = 900.GB

}


singularity {

enabled = true

  // Singularity configs, CHANGE THEM TO YOUR USED FILESYSTEM, if not properly set
the container won't see your files

runOptions = "-B /home -B /dpool"

// where should the containers be downloaded to

cacheDir = "/dpool/ewacker/metagenomics/TOFU-MAaPO/quickstart/singularity_cache"

}
```

Supplementary Notes

Performing a benchmark between TOFU-MAaPO, ATLAS, metaFun and nf-core/mag

For the first use of the pipelines, the user must create a configuration or profile, so that Nextflow uses already installed data bases and adopts to the local conditions of the computer. Both pipelines provide a tutorial for the configuration. This step must be done only once, and the configuration file can be reused for every future run of the respective pipeline. This step must be done only once, and the configuration file can be reused for every future run of the respective pipeline. The used configurations can be found below.

After preparation of a configuration file for our test system for both pipelines and download of the to be used human host genome in a bowtie2 [2] index format and GTDB-Database [3, 4] for both pipelines we could start a workflow comparison.

As nf-core/mag does not feature a direct download feature for samples from SRA by ID, we downloaded the files manually to local storage. After this, we had to create for the 100 samples a CSV-File that provides the IDs and the exact file paths for the downloaded gzipped FASTQ files. After this additional preparation step, we started the nf-core/mag version 3.0.2 with following command:

```
nextflow run nf-core/mag \
   -profile singularity \
   --input /dpool/ewacker/metagenomics/sample_list_raw_nfcore.csv \
   --outdir SRP102150 \
   --host_genome GRCh38 \
   --binqc_tool checkm \
```

```
    --refine_bins_dastool \
    --skip_prokka \
    --postbinning_input refined_bins_only \
    --skip_spades \
    --skip_prodigal \
    --skip_metaeuk \
    -c custom.config \
    -resume
```

We reused the earlier downloaded metagenomes files for the execution of ATLAS [5]. ATLAS was initiated with its 'atlas init –db-dir databases /path/to/metagenomes' command and it created automatically a table for the sample input plus a configuration file with recommended parameters.

In comparison, TOFU-MAaPO was directly executed with a single command call:

```
nextflow run ikmb/TOFU-MAaPO \
    --sra "SRP102150" \
    --assembly \
    --publish_rawbins \
    --publish_megahit \
    -profile biomedinf \
    --apikey **SECRET**\
    --genome human \
    --outdir SRP102150 \
```

```
-work-dir work_SRP102150 \

-c tofu.config
```

The command line call activated the modules numbered 0, 1 and 4 in Supplementary Table 1. The pipeline directly downloaded the samples automatically, which removed the need for manual input-file curation, before starting the pipeline.

We also reused the earlier downloaded metagenomes files for the execution of metaFun[6]. We installed metaFun via conda with the command ‘conda create -c bioconda -c conda-forge -n metafun bioconda::metafun’. The pipeline required an initialization next step to download all required databases and software containers. After successful initialization, the pipeline could be activated by enabling the conda environment. For the benchmark, the required computations are split in metaFun in three modules that needed to be called sequentially: “RAWREAD_QC”, “ASSEMBLY_BINNING”, and “BIN_ASSESSMENT”.

Replication of published results of the NCBI SRA public dataset SRP102150

Further, we want to show the ease to replicate study results with TOFU-MAaPO. We reutilized the same public dataset as before (SRP102150) [7] for which we got basic case/control description through the curatedMetagenomicData Bioconductor R package [8] Version 3.8.0 and started TOFU-MAaPO with a single command using the SRA ID as an input parameter and additionally enabled this time the modules for HUMAnN (v3.6) [9], MetaPhlAn (v4.1) [10] and used as QC tool FASTP [11] instead of the assembly module. Here, we utilize assembly-free tools to estimate the taxonomical abundance with MetaPhlAn and analyze the presence of metabolic pathways with HUMAnN. In the original study of this dataset, Nagy-Szakal et al. used earlier versions of those tools. Therefore, we expected to find similar results in our replication. The pipeline produced abundance tables which the user can then use to analyze the data further.

The TOFU-MAaPO was called with the command on a high-performance cluster to produce the merged abundance tables for further manual analysis of taxonomic abundance and metabolic/pathway abundance:

```
nextflow run ikmb/TOFU-MAaPO -r 1.5.0 --sra SRP102150 --apikey *SECRET* --humann --metaphlan --fastp --genome human
```

Analysis of the pipeline results was performed in R (Version 4.3.1). The merged abundance table produced by MetaPhlAn was converted and merged with metadata into a single phyloseq [12] object, which was further analyzed for differential abundance by phenotype with Maaslin2 [13]. Sex and case-control-status were used as fixed effects. The merged pathway abundance table output of HUMAnN3 was statistically analyzed with Maaslin2. The four most significant metabolic terms were visualized. Visualizations were created with ggplot2 [14].

The merged MetaPhlAn output for the dataset contained entries for 1167 species, while the merged HUMAnN output for pathway abundance contained over 17500 entries. Further analysis showed that we could not detect dramatic shifts in the taxonomic abundances

between the case/control phenotypes (Supplementary Figure 1a). We could not detect significant shifts in the abundance of dominating phyla such as Bacteroidetes, Firmicutes or Proteobacteria. A table of differential abundant taxa on genus level can be found in the Supplementary Table 3. We detected an overlap of controls and ME/CFS patients in a Non-metric Multi-dimensional Scaling plot based on species level Bray-Curtis dissimilarity (Supplementary Figure 1b), in detail, controls show a more stable microbiome signature, while cases where more variable and scattered further than the controls. Additionally, we could also identify WY.5690_TCA_cycle_II_.plants_and_fungi as significantly altered metabolic pathway, while other pathways did not show significant shifts after multiple testing correction after Benjamini–Hochberg (Supplementary Figure 1c). The full Maaslin2 results table for differential metabolic pathway abundance can be found in Supplementary Table 4.

We made all analysis benchmark scripts available at https://github.com/ikmb/TOFUpaper for the reproducibility of our results.

Supplementary Tables

Supplementary Table 1: Software tools implemented within TOFU-MAaPO Version 1.5.0

Module Nr.	Module Name	Tool	Function	Notes
**0**	Input Aggregation	Curl [15]	Download FASTQ files from SRA	
**1**	QC			
		FastQC [16]	Quality Assessment	

			Pre- and Post-QC	
		MultiQC [17]	QC Report	
		BBDuk [18]	Artifact and Phix removal, Read trimming	
		fastp [11]	Read trimming, Adapter removal	
		Bowtie2 [2]	Host reads removal	
**2**	Pathway abundance estimation			
		HUMAnN3 [9]	Estimation of pathways, gene content, coverage	DB needed, can be automatised downloaded
**3**	Taxonomic abundance estimation			
		MetaPhlAn4 [10]	Taxonomical assignment	DB needed, can be automatized downloaded
		Salmon [19]	Taxonomical assignment	DB needed
		Kraken2 [20]	Taxonomical assignment	DB needed
		Bracken [21]	Bayesian reestimation of Kraken2 results	Bracken-ready Kraken2 DB needed
**4**				

		Megahit [22]	Contig Assembler	
		Python3 [23]	Contig size filter	
		minimap2 [24]	Creation of contig catalogue and index	
		SemiBin2 [25]	Binning Tool	
		Vamb [26]	Binning Tool	
		MaxBin2 [27]	Binning Tool	
		MetaBat2 [28]	Binning Tool	
		CONCOCT [29]	Binning Tool	
		Prodigal [30]	Open Reading Frame detection	
	Metagenome assembled genomes	Hmmsearch [31, 32]	Protein sequence annotation with GTDB-TK R207 marker genes	
		MAGScoT [33]	Bin quality scoring and refinement	
		CheckM [34, 35]	Bin Quality Assessment	
		GTDB-Tk [3, 4]	Taxonomical classification of bins	DB needed, can be downloaded with TOFU-MAaPO

Supplementary Figures

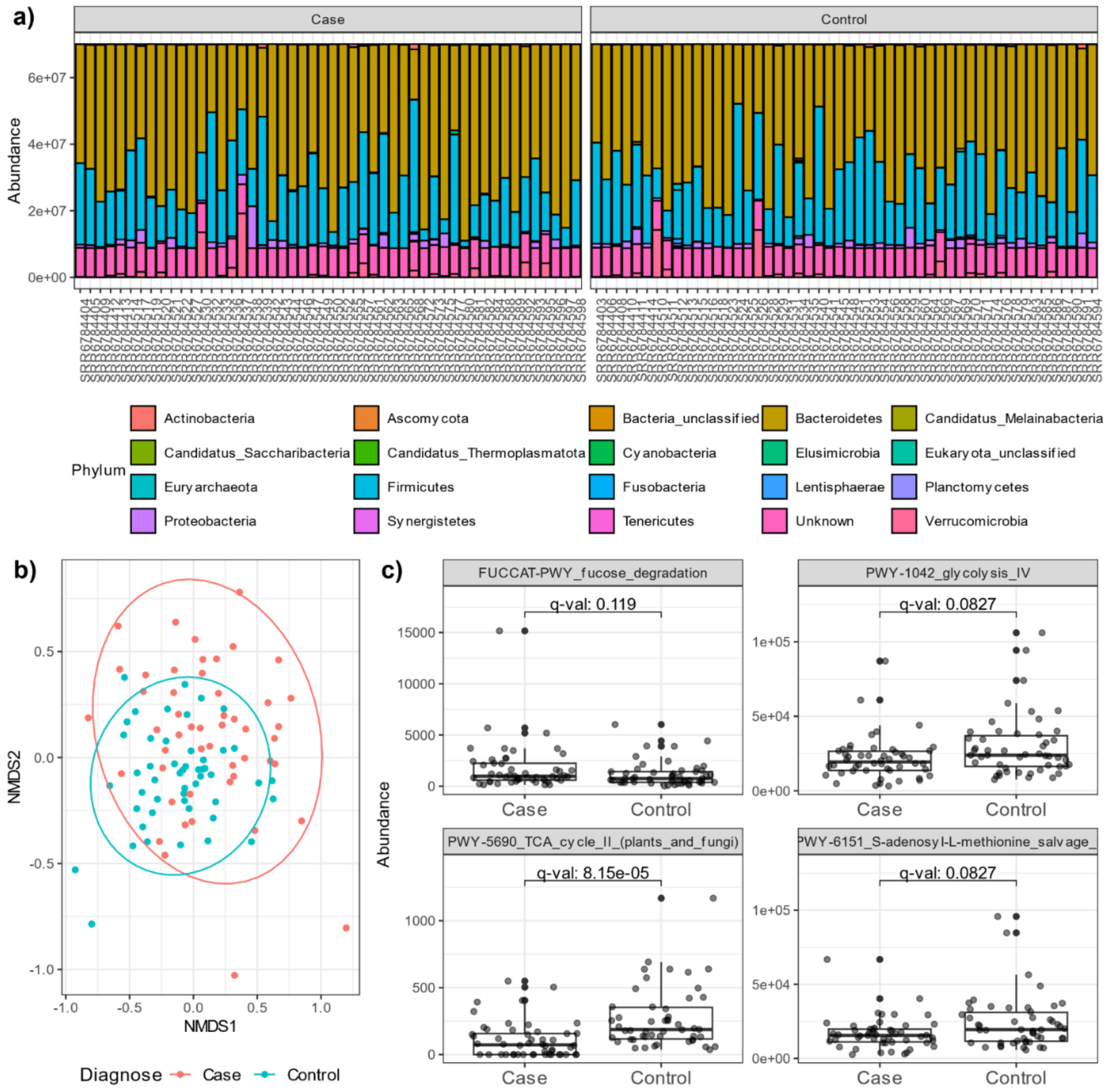

Supplementary Figure 1: Analysis of samples belonging to SRP102150. The dataset contains gut metagenomes of 50 healthy individuals and 50 patients suffering from ME/CFS and were compared in this analysis based on taxonomical and metabolic/gene content. A: MetaPhlAn results for taxonomical composition on phylum level. B: Non-metric multidimensional scaling (NMDS) of Bray-Curtis results on species level based on MetaPhlAn results. C: Four most significant differential pathway abundances between healthy controls and ME/CFS patients. Pathway abundance was calculated with HUMAnN. MaAsLin2 was used for statistical testing.

References

1. Krakau S, Straub D, Gourlé H, Gabernet G and Nahnsen S. nf-core/mag: a best-practice pipeline for metagenome hybrid assembly and binning. NAR Genom Bioinform. 2022;4 1:lqac007. doi:10.1093/nargab/lqac007.

2. Langmead B and Salzberg SL. Fast gapped-read alignment with Bowtie 2. Nat Methods. 2012;9 4:357-9. doi:10.1038/nmeth.1923.

3. Chaumeil PA, Mussig AJ, Hugenholtz P and Parks DH. GTDB-Tk v2: memory friendly classification with the genome taxonomy database. Bioinformatics. 2022;38 23:5315-6. doi:10.1093/bioinformatics/btac672.

4. Chaumeil PA, Mussig AJ, Hugenholtz P and Parks DH. GTDB-Tk: a toolkit to classify genomes with the Genome Taxonomy Database. Bioinformatics. 2019;36 6:1925-7. doi:10.1093/bioinformatics/btz848.

5. Kieser S, Brown J, Zdobnov EM, Trajkovski M and McCue LA. ATLAS: a Snakemake workflow for assembly, annotation, and genomic binning of metagenome sequence data. BMC Bioinformatics. 2020;21 1:257. doi:10.1186/s12859-020-03585-4.

6. Lee HG, Song JY, Yoon J, Chung Y, Kwon S-K and Kim JF. metaFun: An analysis pipeline for metagenomic big data with fast and unified functional

searches. Gut Microbes. 2026;18 1:2611544. doi:10.1080/19490976.2025.2611544.

7. Nagy-Szakal D, Williams BL, Mishra N, Che X, Lee B, Bateman L, et al. Fecal metagenomic profiles in subgroups of patients with myalgic encephalomyelitis/chronic fatigue syndrome. Microbiome. 2017;5 1:44. doi:10.1186/s40168-017-0261-y.

8. Pasolli E, Schiffer L, Manghi P, Renson A, Obenchain V, Truong DT, et al. Accessible, curated metagenomic data through ExperimentHub. Nat Methods. 2017;14 11:1023-4. doi:10.1038/nmeth.4468.

9. Beghini F, McIver LJ, Blanco-Míguez A, Dubois L, Asnicar F, Maharjan S, et al. Integrating taxonomic, functional, and strain-level profiling of diverse microbial communities with bioBakery 3. Elife. 2021;10 doi:10.7554/eLife.65088.

10. Blanco-Míguez A, Beghini F, Cumbo F, McIver LJ, Thompson KN, Zolfo M, et al. Extending and improving metagenomic taxonomic profiling with uncharacterized species using MetaPhlAn 4. Nat Biotechnol. 2023;41 11:1633-44. doi:10.1038/s41587-023-01688-w.

11. Chen S, Zhou Y, Chen Y and Gu J. fastp: an ultra-fast all-in-one FASTQ preprocessor. Bioinformatics. 2018;34 17:i884-i90. doi:10.1093/bioinformatics/bty560.

12. McMurdie PJ and Holmes S. phyloseq: an R package for reproducible interactive analysis and graphics of microbiome census data. PLoS One. 2013;8 4:e61217. doi:10.1371/journal.pone.0061217.

13. Mallick H, Rahnavard A, McIver LJ, Ma S, Zhang Y, Nguyen LH, et al. Multivariable association discovery in population-scale meta-omics studies. PLoS Comput Biol. 2021;17 11:e1009442. doi:10.1371/journal.pcbi.1009442.

14. Hadley W. ggplot2: Elegant Graphics for Data Analysis. Journal of the Royal Statistical Society Series A: Statistics in Society. 2016;174 1:245-6.

15. Hostetter M, Kranz DA, Seed C, Terman C and Ward S. Curl: a gentle slope language for the Web. World wide web journal. 1997;2 2:121-34.

16. FastQC (2012) Andrews S, Krueger F, Segonds-Pichon A, Biggins L, Krueger C and Wingett S (Version 0.11.9) https://github.com/s-andrews/FastQC/releases/tag/v0.11.9.

17. Ewels P, Magnusson M, Lundin S and Käller M. MultiQC: summarize analysis results for multiple tools and samples in a single report. Bioinformatics. 2016;32 19:3047-8. doi:10.1093/bioinformatics/btw354.

18. BBDuk (2022) Bushnell B (Version 39.00) https://sourceforge.net/projects/bbmap/

19. Patro R, Duggal G, Love MI, Irizarry RA and Kingsford C. Salmon provides fast and bias-aware quantification of transcript expression. Nat Methods. 2017;14 4:417-9. doi:10.1038/nmeth.4197.

20. Shaw J and Yu YW. Rapid species-level metagenome profiling and containment estimation with sylph. Nat Biotechnol. 2024; doi:10.1038/s41587-024-02412-y.

21. Lu J, Breitwieser FP, Thielen P and Salzberg SL. Bracken: estimating species abundance in metagenomics data. PeerJ Computer Science. 2017;3:e104. doi:10.7717/peerj-cs.104.

22. Li D, Liu CM, Luo R, Sadakane K and Lam TW. MEGAHIT: an ultra-fast single-node solution for large and complex metagenomics assembly via succinct de Bruijn graph. Bioinformatics. 2015;31 10:1674-6. doi:10.1093/bioinformatics/btv033.

23. Van Rossum G and Drake FL, Jr. Python tutorial. Centrum voor Wiskunde en Informatica Amsterdam, The Netherlands; 1995.

24. Li H. Minimap2: pairwise alignment for nucleotide sequences. Bioinformatics. 2018;34 18:3094-100. doi:10.1093/bioinformatics/bty191.

25. Pan S, Zhao XM and Coelho LP. SemiBin2: self-supervised contrastive learning leads to better MAGs for short- and long-read sequencing. Bioinformatics. 2023;39 39 Suppl 1:i21-i9. doi:10.1093/bioinformatics/btad209.

26. Nissen JN, Johansen J, Allesøe RL, Sønderby CK, Armenteros JJA, Grønbech CH, et al. Improved metagenome binning and assembly using deep variational autoencoders. Nat Biotechnol. 2021;39 5:555-60. doi:10.1038/s41587-020-00777-4.

27. Wu YW, Simmons BA and Singer SW. MaxBin 2.0: an automated binning algorithm to recover genomes from multiple metagenomic datasets. Bioinformatics. 2016;32 4:605-7. doi:10.1093/bioinformatics/btv638.

28. Kang DD, Li F, Kirton E, Thomas A, Egan R, An H, et al. MetaBAT 2: an adaptive binning algorithm for robust and efficient genome reconstruction from metagenome assemblies. PeerJ. 2019;7:e7359. doi:10.7717/peerj.7359.

29. Alneberg J, Bjarnason BS, de Bruijn I, Schirmer M, Quick J, Ijaz UZ, et al. Binning metagenomic contigs by coverage and composition. Nat Methods. 2014;11 11:1144-6. doi:10.1038/nmeth.3103.

30. Hyatt D, Chen GL, Locascio PF, Land ML, Larimer FW and Hauser LJ. Prodigal: prokaryotic gene recognition and translation initiation site identification. BMC Bioinformatics. 2010;11:119. doi:10.1186/1471-2105-11-119.

31. Mistry J, Finn RD, Eddy SR, Bateman A and Punta M. Challenges in homology search: HMMER3 and convergent evolution of coiled-coil regions. Nucleic Acids Res. 2013;41 12:e121. doi:10.1093/nar/gkt263.

32. Johnson LS, Eddy SR and Portugaly E. Hidden Markov model speed heuristic and iterative HMM search procedure. BMC Bioinformatics. 2010;11:431. doi:10.1186/1471-2105-11-431.

33. Rühlemann MC, Wacker EM, Ellinghaus D and Franke A. MAGScoT: a fast, lightweight and accurate bin-refinement tool. Bioinformatics. 2022;38 24:5430-3. doi:10.1093/bioinformatics/btac694.

34. Parks DH, Imelfort M, Skennerton CT, Hugenholtz P and Tyson GW. CheckM: assessing the quality of microbial genomes recovered from isolates, single cells, and metagenomes. Genome Res. 2015;25 7:1043-55. doi:10.1101/gr.186072.114.

35. Chklovski A, Parks DH, Woodcroft BJ and Tyson GW. CheckM2: a rapid, scalable and accurate tool for assessing microbial genome quality using machine learning. Nat Methods. 2023;20 8:1203-12. doi:10.1038/s41592-023-01940-w.
