# Supplementary material for: TOFU-MAaPO: fast, scalable and reproducible analysis of large metagenome sequence data from the Sequence Read Archive
Source: Nat Commun. 2026 Jun 11;17:5215. doi: 10.1038/s41467-026-74033-9 (PMC13260335; doi:10.1038/s41467-026-74033-9)
Supplement: Supplementary file 2 — Reporting Summary [file 41467_2026_74033_MOESM2_ESM.pdf]

nature portfolio

Corresponding author(s): David Ellinghaus

Last updated by author(s): Mar 24, 2025

Reporting Summary

Nature Portfolio wishes to improve the reproducibility of the work that we publish. This form provides structure for consistency and transparency in reporting. For further information on Nature Portfolio policies, see our Editorial Policies and the Editorial Policy Checklist.

Statistics

For all statistical analyses, confirm that the following items are present in the figure legend, table legend, main text, or Methods section.

n/a	Confirmed	
☐	☒	The exact sample size (*n*) for each experimental group/condition, given as a discrete number and unit of measurement
☐	☒	A statement on whether measurements were taken from distinct samples or whether the same sample was measured repeatedly
☐	☒	The statistical test(s) used AND whether they are one- or two-sided<br>*Only common tests should be described solely by name; describe more complex techniques in the Methods section.*
☐	☒	A description of all covariates tested
☐	☒	A description of any assumptions or corrections, such as tests of normality and adjustment for multiple comparisons
☐	☒	A full description of the statistical parameters including central tendency (e.g. means) or other basic estimates (e.g. regression coefficient) AND variation (e.g. standard deviation) or associated estimates of uncertainty (e.g. confidence intervals)
☐	☒	For null hypothesis testing, the test statistic (e.g. *F*, *t*, *r*) with confidence intervals, effect sizes, degrees of freedom and *P* value noted<br>*Give P values as exact values whenever suitable.*
☒	☐	For Bayesian analysis, information on the choice of priors and Markov chain Monte Carlo settings
☒	☐	For hierarchical and complex designs, identification of the appropriate level for tests and full reporting of outcomes
☐	☒	Estimates of effect sizes (e.g. Cohen's *d*, Pearson's *r*), indicating how they were calculated

Our web collection on statistics for biologists contains articles on many of the points above.

Software and code

Policy information about availability of computer code

Data collection	TOFU-MAaPO is open-source and freely available as a containerized Nextflow pipeline at https://github.com/ikmb/TOFU-MAaPO .
Data analysis	All analysis benchmark scripts are available at https://github.com/ikmb/TOFUpaper .

For manuscripts utilizing custom algorithms or software that are central to the research but not yet described in published literature, software must be made available to editors and reviewers. We strongly encourage code deposition in a community repository (e.g. GitHub). See the Nature Portfolio guidelines for submitting code & software for further information.

Data

Policy information about availability of data

All manuscripts must include a data availability statement. This statement should provide the following information, where applicable:

- Accession codes, unique identifiers, or web links for publicly available datasets
- A description of any restrictions on data availability
- For clinical datasets or third party data, please ensure that the statement adheres to our policy

Benchmark dataset #1: original data available at https://www.ncbi.nlm.nih.gov/Traces/study/?acc=SRP102150&o=acc_s%3Aa Benchmark dataset #2: original data available at https://www.bioconductor.org/packages/release/data/experiment/html/curatedMetagenomicData.html see individual list of samples used at https://github.com/ikmb/TOFUpaper/blob/master/curatedmetagenomics_sample_list_sex_age.csv

Research involving human participants, their data, or biological material

Policy information about studies with human participants or human data. See also policy information about sex, gender (identity/presentation), and sexual orientation and race, ethnicity and racism.

Reporting on sex and gender	Benchmark dataset #1: We used a publicly available dataset of 100 faecal metagenomes from myalgic encephalomyelitis/ chronic fatigue syndrome patients (ME/CFS) and controls stored in the NCBI Sequence Read Archive with ID SRP102150. Females: 82; Males: 18 Case/contol status and information about sex is available at https://www.ncbi.nlm.nih.gov/Traces/ study/?acc=SRP102150&o=acc_s%3Aa Cases included 41 female and 9 male ME/CFS patients (mean age 51.1 years; standard error of the mean (SEM) 1.6). Controls included 41 female and 9 male subjects (mean age 51.3 years; SEM 1.6). Benchmark dataset #2: 4969 females, 4811 males, unknown 6712 see individual list of sex and age at https://github.com/ ikmb/TOFUpaper/blob/master/curatedmetagenomics_sample_list_sex_age.csv
Reporting on race, ethnicity, or other socially relevant groupings	Benchmark dataset #1: 50 ME/CFS patients and 50 healthy controls were frequency-matched for age, sex, race/ethnicity, geographic site, and season of sampling, see original publication Nagy-Szakal et al. Microbiome 2017, https:// pubmed.ncbi.nlm.nih.gov/28441964/ Benchmark dataset #2: potentially information available from original studies, see PMIDs in https://github.com/ikmb/TOFUpaper/blob/master/curatedmetagenomics_sample_list_sex_age.csv
Population characteristics	Benchmark dataset #1: 50 ME/CFS patients and 50 healthy controls were frequency-matched for age, sex, race/ethnicity, geographic site, and season of sampling, see original publication Nagy-Szakal et al. Microbiome 2017, https:// pubmed.ncbi.nlm.nih.gov/28441964/ Benchmark dataset #2: mean age females: 29.3; mean age males: 21.1; potentially more information available from original studies, see PMIDs in https://github.com/ikmb/TOFUpaper/blob/master/ curatedmetagenomics_sample_list_sex_age.csv
Recruitment	Benchmark dataset #1: Subjects included 50 ME/CFS cases and 50 healthy controls recruited at four sites across the USA (New York, NY; Salt Lake City, UT; Incline Village, NV; and Miami, FL) who met the 1994 CDC Fukuda and the 2003 Canadian consensus criteria for ME/CFS, see original desription in Nagy-Szakal et al. Microbiome 2017, https:// pubmed.ncbi.nlm.nih.gov/28441964/ Benchmark dataset #2: information available from original studies, see PMIDs in https://github.com/ikmb/TOFUpaper/blob/master/curatedmetagenomics_sample_list_sex_age.csv
Ethics oversight	Benchmark dataset #1: All participants provided informed written consent in accordance with protocols approved by the Institutional Review Board at Columbia University Medical Center, see Nagy-Szakal et al. Microbiome 2017, https:// pubmed.ncbi.nlm.nih.gov/28441964/ Benchmark dataset #2: information available from original studies, see PMIDs in https://github.com/ikmb/TOFUpaper/blob/master/curatedmetagenomics_sample_list_sex_age.csv

Note that full information on the approval of the study protocol must also be provided in the manuscript.

Field-specific reporting

Please select the one below that is the best fit for your research. If you are not sure, read the appropriate sections before making your selection.

☒ Life sciences ☐ Behavioural & social sciences ☐ Ecological, evolutionary & environmental sciences

For a reference copy of the document with all sections, see nature.com/documents/nr-reporting-summary-flat.pdf

Life sciences study design

All studies must disclose on these points even when the disclosure is negative.

Sample size	Benchmark dataset #1: we used a publicly available dataset of 100 faecal metagenomes from myalgic encephalomyelitis/chronic fatigue syndrome patients and controls stored in the NCBI Sequence Read Archive with ID SRP102150. Case/contol status and information about sex is available at https://www.ncbi.nlm.nih.gov/Traces/study/?acc=SRP102150&o=acc_s%3Aa 2) Benchmark dataset #2: n=16,492 see https://github.com/ikmb/TOFUpaper/blob/master/curatedmetagenomics_sample_list_sex_age.csv
Data exclusions	Benchmark dataset #1: no data exclusions Benchmark dataset #2 (see Methods in Manuscript): The sample annotation was taken from the ExperimentHub database via the Bioconductor package curatedMetagenomicData version 3.8.0 and uses SRA IDs. If multiple SRA run IDs belonged to the same sample SRA ID in ExperimentHub, only the first listed SRA run ID was used (in longitudinal studies, SRA run IDs of different time points are assigned to the same SRA sample IDs). In total, 17,934 SRA run IDs from this source were taken and 17,090 run IDs could be successfully automatically queried from the SRA via TOFU-MAaPO and their data downloaded. Taxonomic composition analysis with Sylph yielded non-empty results for 16,492 SRA run IDs.
Replication	Benchmark dataset #1 was originally published by Nagy-Szakal et al. Microbiome 2017, https://pubmed.ncbi.nlm.nih.gov/28441964/ and validation results are shown in the Supplementary Results section and Supplementary Figure 1. Benchmark dataset #2: not applicable
Randomization	Not applicable
Blinding	Not applicable

Reporting for specific materials, systems and methods

We require information from authors about some types of materials, experimental systems and methods used in many studies. Here, indicate whether each material, system or method listed is relevant to your study. If you are not sure if a list item applies to your research, read the appropriate section before selecting a response.

Materials & experimental systems

n/a	Involved in the study
☒	☐ Antibodies
☒	☐ Eukaryotic cell lines
☒	☐ Palaeontology and archaeology
☒	☐ Animals and other organisms
☒	☐ Clinical data
☒	☐ Dual use research of concern
☒	☐ Plants

Methods

n/a	Involved in the study
☒	☐ ChIP-seq
☒	☐ Flow cytometry
☒	☐ MRI-based neuroimaging

Plants

Seed stocks

Report on the source of all seed stocks or other plant material used. If applicable, state the seed stock centre and catalogue number. If plant specimens were collected from the field, describe the collection location, date and sampling procedures.

Novel plant genotypes

Describe the methods by which all novel plant genotypes were produced. This includes those generated by transgenic approaches, gene editing, chemical/radiation-based mutagenesis and hybridization. For transgenic lines, describe the transformation method, the number of independent lines analyzed and the generation upon which experiments were performed. For gene-edited lines, describe the editor used, the endogenous sequence targeted for editing, the targeting guide RNA sequence (if applicable) and how the editor was applied.

Authentication

Describe any authentication procedures for each seed stock used or novel genotype generated. Describe any experiments used to assess the effect of a mutation and, where applicable, how potential secondary effects (e.g. second site T-DNA insertions, mosiacism, off-target gene editing) were examined.
