# Supplementary material for: TOFU-MAaPO: fast, scalable and reproducible analysis of large metagenome sequence data from the Sequence Read Archive
Source: Nat Commun. 2026 Jun 11;17:5215. doi: 10.1038/s41467-026-74033-9 (PMC13260335; doi:10.1038/s41467-026-74033-9)
Supplement: Supplementary file 3 — Transparent Peer Review file [file 41467_2026_74033_MOESM3_ESM.pdf]

nature portfolio

Peer Review File

TOFU-MAaPO: Fast, scalable and reproducible analysis of large metagenome sequence data from the Sequence Read Archive

Corresponding Author: Professor David Ellinghaus

This file contains all reviewer reports in order by version, followed by all author rebuttals in order by version.

Version 0:

Reviewer comments:

Reviewer #1

(Remarks to the Author)
There are six advancements that mark an inflection point in bioinformatics, and scientific computing more generally :

* Developing and maintaining code under distributed revision control
* Organizing software development with social programming systems
* Distributing software and its dependencies using packages managers
* Deploying software in encapsulated environments (i.e., containers)
* Coordinating execution with formal workflow languages
* Analyzing results in literate programming environments

These technologies are deeply interpenetrating, and when used together, produce multiplicative benefits to the scientific process. As a small example, the trivial effort that was required to download, install, deploy, run and examine the output of this software allowed this reviewer to quickly verify many of the key claims in this manuscript. This is not merely a technical advancement. It is a step forward for scientific inquiry.

```bash
N E X T F L O W ~ version 24.10.5

Launching `https://github.com/ikmb/tofu-maapo` [distraught_mcnulty] DSL2 - revision: 36d8b0b796 [master]


===============================================================================
IKMB | TOFU-MAaPO | Version 1.5.0
===============================================================================
Nextflow Version: 24.10.5
Container Engine: singularity
=======INPUTS==================================================================
Reads: : *_R{1,2}.fastq.gz
Host genome: : false
===============================================================================
Command Line: nextflow run ikmb/tofu-maapo -profile quickstart --reads '*_R{1,2}.fastq.gz' --cleanreads --outdir results
===============================================================================
No Host genome was specified!
[- ] tofumaapo:QC:FASTQC_raw -
[- ] tofumaapo:QC:TRIMREADS -
[- ] tofumaapo:QC:CLEANREADS -
[- ] tofumaapo:QC:COLLECTOR -
executor > local (3)
executor > local (5)
```

```
[4b/6cc43d] tof…o:QC:FASTQC_raw (PSM6XBR1) | 1 of 1 ✔
executor > local (7)
[4b/6cc43d] tof…o:QC:FASTQC_raw (PSM6XBR1) | 1 of 1 ✔
[90/ba325d] tof…po:QC:TRIMREADS (PSM6XBR1) | 1 of 1 ✔
[93/7c9eaf] tof…o:QC:CLEANREADS (PSM6XBR1) | 1 of 1 ✔
[aa/458b6e] tof…po:QC:COLLECTOR (PSM6XBR1) | 1 of 1 ✔
[5f/ad7445] tof…QC:FASTQC_clean (PSM6XBR1) | 1 of 1 ✔
[91/3d5245] tofumaapo:MULTIQC | 1 of 1 ✔
[10/9367f0] tof…aapo:SOFTWARE_VERSIONS (1) | 1 of 1 ✔
==========================================
Duration: 13m 36s
==========================================
Completed at: 21-Apr-2025 13:46:23
Duration : 13m 37s
CPU hours : 0.3
Succeeded : 7
```

The main fault of this manuscript is that the authors *undersell* what they (and others) have achieved, and *understate* the implicit objectives of their own project. As the authors note, several other pipelines exist that, broadly speaking, do the same thing as TOFU-MAaPO. Normally, the introduction of yet another new tool into a crowded space would invite the XKCD 927 critique (https://xkcd.com/927/) : proliferating standards drives fragmentation of the community, thwarts comparative analysis and impedes forward momentum of scientific insight. The authors avoid this problem by making appropriate selections of tools, technologies and paradigms.

I would like to discourage the authors from viewing (and presenting) their software as Yet Another Analytical Pipeline for Metagenomes that competes in a field of similar alternatives. There are a finite number of reasonable analytical pathways from raw reads to annotated MAGs. There are a finite number of reasonable ways to structure those pathways and marshal execution of those tools. There are a finite number of reasonable ways to manage the input, intermediate and final data. Because the authors have elected to develop their software using a distributed revision control platform with integrated social programming features (Github), to use modular, encapsulated deployment environments (Singularity/Apptainer) and a formal workflow language (Nextflow), the result is that TOFU-MAaPO is not competing with ATLAS, MetaWRAP, nf-core/mag, or any of the dozens of other pipelines for metagenome analysis. It is in *conversation* with those projects. Indeed, the authors based it on [an earlier workflow](https://github.com/marchoeppner/metagenomic-profiling), and it has already been forked at least five times. Concepts, implementations, and in many cases major chunks of code and subsystems can be (and often are) freely shared among these projects. There are not dozens of competing tools, but rather there is *one* community striving together to solve a common set of challenges. Tools like Github, Singularity and Nextflow make it practical for the community to have discussions about the merits of different approaches that are rooted in practice and grounded by data. TOFU-MAaPO is a statement within that conversation that welcomes further conversation, and this is a Very Good Thing. It is a mature thing. It is a responsible and healthy thing.

Of course, this is just my opinion. However, I suspect that the authors are very likely motivated by thoughts of a very similar nature. I would like to see those ideas centered a more clearly and stated more forcefully in the final version of this manuscript. As a community, I think it is long past time for us to come together and see what our combined efforts have yielded, and I wholeheartedly applaud the authors contribution to that effort.

Technical points

I have only one major criticism of the software itself. At the moment, TOFU-MAaPO can run either with input from SRA or with input from local FASTQ files, but not a combination of both. This is a very unfortunate limitation. In almost all cases, a researcher would want to place their data into context with relevant, previously published data. This is best practice, and should be a core feature.

I am not a Nextflow expert, but browsing the codebase does not suggest to me that it would be a major overhaul to make this possible.

Line by line

77 : There may be countless ways to select and combine bioinformatics tools, but there are only a handful of *reasonable* ways in which they can be combined. Containers and workflows do not merely solve a technical problem, they solve a *scientific* problem by structuring discourse among human beings. Containers and workflows convert myriad decisions about inputs, outputs, configurations, versions, and parameters into a single object that can be discussed, criticized, applied, reused and improved upon. The fact that this leads to technical conveniences is a side effect!

94-96 : Too may ideas are stuffed into a sentence that isn't up the task of holding them.

111 : A simple... what, exactly?

126-128 : Please discuss speed and accuracy in separate sentences. The parallel construction is confusing, and maybe a bit misleading depending on how you interpret it.

408 : Please correct the grammar "allows to create."

417 : Is "DAS tool" actually how you're supposed to say it? I'm not sure if it is supposed to be a German pun or not...

450-453 : No! Don't just assume that longer runtimes are are due to poorer parallelization! This is easily testable! If you want people to write better workflows, then please try to find out what the problems actually are!

496 : Metagenomes are countable nouns, so they don't have amounts.

501 : Docker? I thought it was Singularity/Apptainer? There's a docker image for the whole project, though. Is that what you're talking about here?

506 : Typo. "quality or" should be "quality of."

512 : This is not really true. TOFU-MAaPO sits at the beginning of the analytical cycle. Most of the important scientific questions lay downstream from MAGs, taxonomic annotations and functional annotations, and there are many other pipelines and tools the people would want to use after arriving at that point.

517-530 : This final paragraph is bit of a letdown. This software is super useful, and it is going to get more useful, and the things that will allow it to become more useful are exciting and important! The authors clearly understand this, but many of their intended users are focused on biological questions. Such folks may understand how the software works in a technical sense, but may not appreciate the impact these technologies are having on the research process in our field.

611 : Please fix the formatting of the equation in Figure 3. When using absolute value verticals, the parentheses are redundant. The LaTeX code should be something like $\log_{10}{\lvert \frac{\text{Bacteroides}}{\text{Prevotella}}\rvert}$.

Final remarks

I've used some language in this review that could be taken to imply a major rewrite, but but want to be clear that I do not think that is the case. Almost everything amounts to asking the authors to more clearly articulate *why* they made these good decisions. I'd call that a minor revision.

(Remarks on code availability)
This project is very close to what I would consider the Gold Standard in terms of reproducibility, documentation, availability and redistributability. It is actually rather difficult to imagine what more one could ask for without sounding churlish, but here's my wishlist :

[1] The inability to use mixed input from SRA and local files is unfortunate.
[2] More tests, maybe?
[3] More engagement on Github between the authors and other metagenomics workflows, maybe?
[4] More comments in the code
[5] Jupyter Notebook output

Reviewer #2

(Remarks to the Author)
This paper describes an automated workflow (TOFU-MAaPO) based on NetFlow and Singularity/Apptainer for metagenome analysis that encompasses automatic data download, read QC, assembly- and read-level analyses, with the former proceeding through to MAG quality assessment, taxonomic assignment and coverage estimation. In a benchmarking exercise against two comparable workflow products, ATLAS and nf-core/mag. The authors show that their software runs faster than either tested alternative work and imply that their workflow is superior due to the increased number, and quality profile, of the MAG catalogues that are recovered. The software can be installed and run (see below), and as such, the workflow is a useful contribution, particularly as it drops out both MAG and read level taxonomic- and functional-outputs and we would certainly consider using it in our laboratory for routine bulk processing of metagenome data. Having said that, there are major concerns about the second claim made above, and the lack of balance in the presentation.

The second claim referred to above, namely that TOFU-MAaPO “TOFU-MAaPO produced 42% to 77% higher quality MAGs” (line 38). While this is a statement of fact, in scientific terms I do not believe this claim has any meaning. It is simply a consequence of using five binning algorithms, rather than two or three, along with differences induced by different assemblers (SPADes vs MegaHIT), genome quality scoring (CheckM vs. CheckM2), dereplication and selection procedures (DasTools vs. MAGScoT) and MAG reporting criteria. This results from the fact that there are non-overlapping sets of MAG recovered by each method, and so, of course, you will see more MAGs if you incorporate more methods into an analysis (this can ascertained easily by making a presence-absence matrix of the non-redundant MAGs observed per workflow, using a suitable categorisation (e.g. secondary cluster in dRep). We do appreciate that the authors have taken effort to standardize the comparison as much as possible, but this is not a “benchmark” in any meaningful sense, because TOFU-MAaPO is inherently pre-biased to giving a better readout.

On the technical systems level, this paper is presenting an overly optimistic picture of the utility of these tools in practice: specifically, their installation is often very system specific. For example, we tried to install and run TOFU-MAaPO on two HPC systems we use for our routine work on metagenomics; on one, the sys admin does not permit Singularity to be installed, even as a local installation. On the other, Singularity can be run, but external downloads are not permitted. We ended up running TOFU-MAaPO on a VirtualBox install of Linux on a Mac desktop machine, and it certainly worked on the example data, but in practical terms, we would not be able to use it on either of the servers for which we have routine access. The other disadvantage with these bundled workflows is that as time goes on, there is often a lot of work needed to locally maintain the installation and operation, due to incompatibilities between new versions of the component software. The Discussion could have provided an informative and balanced view of these and related limitations, rather than simply highlighting the advantages.

(Remarks on code availability)
The software was attempted to be installed on two HPC systems; one did not permit Singularity/Apptainer to the installed, while the other did not permit automatic downloaded. We tested the workflow (MAG analysis only) on Linux via VirtualBox installed on a Mac desktop machine, with confirmed operation.

Version 1:

Reviewer comments:

Reviewer #1

(Remarks to the Author)
The authors have addressed all substantive points raised in my previous review. I am particularly pleased that they've implemented the ability to mix SRA records and local FASTQ files in an analysis.

In light of the difficulties encountered by Reviewer 2, I tried running TOFU-MAaPO in a more restricted zone of our institute's computer cluster, where I did encounter a minor annoyance.

The issue is an unfortunate side effect of TOFU-MAaPO's single-command design, which aims to perform data retrieval and data processing concurrently controlled by the same command. This is, I think, the correct design philosophy, but unfortunately it won't always work smoothly in compute environments where compute resources and network access are partitioned by policy. Many (most?) HPC environments restrict public internet access from compute nodes. However, the software's error reporting and restartability allow users to work around the limitations of their compute environment if the workflow halts for this reason.

I think this is more of an documentation issue than a technical deficiency. I'm not a nextflow expert, but I think it is possible to use ``-entry`` or ``-stub-run`` to work within policy limitations fetch the data and pull the required containers. The user would marshal the data and containers on the head node of their HPC, and then dispatch the pipeline to compute nodes without public internet access. Some of the advantages of concurrent data retrieval and processing could be recovered by splitting the run into batches. Indeed, the authors describe this strategy in the manuscript!

Ideally, users should not have to be Nextflow programmers to use this software. I encourage the authors anticipate and document common use cases that require the user to modify the workflow execution flow. For very common situations (like not having internet access on compute nodes), it's probably a good idea to wrap the needed Nextflow arguments in a custom argument, instead of relying on generic workflow semantics.

My only substantive request this time around is to explicitly address this use case in the documentation. Please give the section in the documentation a title that a frustrated user would recognize as a solution!

(Very) minor points

There are a few turns of phrase in the manuscript that, while understandable, are a bit awkward. Some of these seem to come from applying Nextflow's rather peculiar use of specific words (e.g., "execution" and "implement") to general prose. There are also a few strange or incorrect constructions (e.g., "that allows to continue at a later date," line 164). I will list the ones I see, but it might be a good idea for the manuscript to get a brush-up by a copy editor before it goes to press.

L. 63 : I don't quite understand what is meant by putting "clinical" is in parentheses. Phenotypes that may or may not be phenotypes of the host rather than the organism itself?

L. 85, 87 : Grammatically, I think "executions" should probably be singular, but this is Nextflow's jargon for something like process, instance, task, or something like that. To me, using "execution" as a noun always sounds like a reference to the death penalty. Please find a more generally used term.

L. 99 : "lacks the use of" -> "does not use"

L. 101 : "...assembly and binning based that fulfills..." Looks like some kind of copy editing error?

L. 164 : "...that that allows to continue at a later point..." How about, "Nextflow workflows use automatic caching, and can be resumed after interruption from the last completed step. This feature can be leveraged feed output from previously completed runs into modules that were not included in the initial analysis, permitting users to perform additional analysis without starting the workflow from the beginning."

L. 243 : Phix should be written phi X, Phi X or ΦX174.

L. 325 : Implemented is not quite the right word (I think this is another bit of Nextflow jargon). I would say "packaged," "integrated," "adapted," or something like that.

L. 388-391 : Suggested rephrase : For benchmarking, we used SRA project SRP102150, which has 100 SRA IDs. While TOFU-MAaPO can download and preprocess data from SRA itself (see Methods), both ATLAS and nf-core/mag required these IDs to be manually downloaded and staged. It was also necessary to generate a CSV file containing file path information for ng-core/mag.

L. 403-404 : "...with a similar sample size led resulted in exceeding the available disk space..." -> "Because the default co-binning setting in the previous test exceeded our 3Tb limit for disk space, we elected to use single binning for ATLAS."

L. 409 : "in order not to give TOFU-MAaPO an unfair speed advantage over ATLAS, which also uses FastWC and BBDuk, although nf-core/mag uses the much faster fastp tool." I'm not sure why these confusing caveats are included. Just say which trimming method was used in each case, and mention separately what alternatives were available.

L. 442-444 : Rephrase in active voice.

L. 459-465 : Thank you for pinpointing the root cause of the long run time in nf-core/mag. The phrasing is confusing, though. Is this what you mean? -> "The root cause of this surprisingly long run time is evidently due to the way the CONCOCT binning tool is applied within the nf-core/mag workflow, which results in a high overhead from spawning large numbers of utility processes (e.g., compression and decompression). The CONCOCT module in TOFU-MAaPO achieves substantially better performance (2.23 hours on with eight cores per sample verses 8.38 hours with on twelve cores per sample) by leveraging a more optimized parallelization strategy."

L. 470-473 : Proposed rephrase : ATLAS produced about half as many MAGs (XXX vs. YYY), using three fewer binning tools and a slightly longer running time.

L. 504 : "without having to go through" -> "without the need to perform"

L. 517 : Suggest "application of," "architecture," or "synthesis" rather than "implementation." The implementation belongs to the respective authors of those binning tools.

L. 519-522 : The reason you get better results by integrating the results from five different binning methods is probably more subtle (and more interesting) than this. There are lots of papers on combining the results of multiple binners, and folks have been tinkering with this concept for at least 20 years. Just cite some of your favorites. Some recent papers :

- https://www.nature.com/articles/s41467-025-57957-6
- https://www.nature.com/articles/s41579-023-00981-4
- https://enviromicro-journals.onlinelibrary.wiley.com/doi/10.1111/1462-2920.16351

L. 531 : "...to keep up to date for processing and analyzing metagenomic data." The first half of this sentence is sufficient. You can delete this part.

L. 534 : delete "still"

L. 536 - 539 : Just say, "We are planning to add additional assembly tools and binning tools to TOFO-MAaPU."

L. 543 : I don't mind the word "equip," but it's probably better to be consistent about what verb you use mean "adding a new piece of software to the workflow."

L. 545 - 549 : The fact that this workflow enables small research groups to perform analysis that previously required the labor of large consortia is a SUPER IMPORTANT POINT! I know this from years of personal experience working in the trenches of exactly these kinds of consortia. Better tooling isn't just a nice-to-have; better tooling enables better science. Please add it to the abstract!

(Remarks on code availability)
My only substantive request is to add a short section in the documentation that explicitly address the use case where container pulls and data retrieval are performed separately. This is a use case that many HPC users will not realize they need until they encounter errors. Please give the section in the documentation a title that a frustrated user would recognize as a solution!

Reviewer #3

(Remarks to the Author)
I have tested the code for TOFU-MAaPO, and I don't doubt the fluency of running it or its expected good performance and results. As commented by both reviewers, providing a good pipeline to integrate all the related tools is the major contribution of TOFU-MAaPO.

Regarding the comment about “TOFU-MAaPO produced 42% to 77% higher quality MAGs” from Reviewer #2, I agree with the reviewer. The performance gain is due to using more binning tools and different refinement tools, not due to the algorithmic innovation of TOFU-MAaPO itself. The statement that “TOFU-MAaPO produced 42% to 77% higher quality MAGs” is scientifically not fair to other pipelines, as they use different assembly, binning, and refinement tools. The authors should at least clarify that the performance gain is not a direct benefit of TOFU-MAaPO, but rather the result of combining different binning tools via MAGScoT -- and this weakens the contribution of TOFU-MAaPO. In addition, contig binning and binning refinement are very competitive areas where "better" tools are constantly being developed. For example, based on our experience in metagenomics, COMEBin is currently the best contig binning tool for producing high-quality MAGs, better than all binning tools used in TOFU-MAaPO. Therefore, an established pipeline such as TOFU-MAaPO must be subject to frequent upgrades to modern tools in order to catch up with the state-of-the-art. This means the steps of the pipeline might be good today, but they could quickly become obsolete tomorrow. This is a limitation of all pipeline tools, so it's good that authors have discussed it in the Discussion.

Regarding the comment about installing TOFU-MAaPO from Reviewer #2, it's worth mentioning that installing Singularity is painful, which was correctly complained about by Reviewer #2, though I don't think this is a critical fault of the software itself.

However, I must point out that the work of TOFU-MAaPO largely overlaps with a recent tool called metaFun (https://doi.org/10.1080/19490976.2025.2611544), which offers even more comprehensive analysis (such as strain-level analysis) and is well maintained and documented (https://metafun-doc.readthedocs.io/en/latest/index.html). metaFun also uses Nextflow but with Apptainer, avoiding the Singularity issues seen in TOFU-MAaPO by Reviewer #2. The manuscript does not even mention metaFun, let alone benchmark against it. This is my major concern. This might be understandable as metaFun was published only 2 months ago, but it does weaken the contribution of TOFU-MAaPO.

Therefore, while I do appreciate the authors' efforts to put these metagenomic analysis tools together into a good pipeline tool, the contribution is not sufficient for publication in Nature Communications. I would only recommend publication if the 1,652 non-redundant species-level genome bins of IBD mentioned in the Introduction were constructed using TOFU-MAaPO and published with this paper, rather than in previous work.

(Remarks on code availability)

Version 2:

Reviewer comments:

Reviewer #1

(Remarks to the Author)
I have no substantive criticisms and am satisfied with the revised manuscript.

I spotted a few minor formatting issues, such as the capitalization of metaFun on line 411. This kind of problem is typically introduced by autocorrect in word processing software, so keep an eye out for formatting of tool names in the final version.

(Remarks on code availability)

Reviewer #3

(Remarks to the Author)
All my concerns have been addressed. The flexibility and extensibility of the pipeline are particularly important in this area, and I appreciate the development of TOFU-MAaPO.

(Remarks on code availability)
The codes are well organized and tested.

Open Access This Peer Review File is licensed under a Creative Commons Attribution 4.0 International License, which permits use, sharing, adaptation, distribution and reproduction in any medium or format, as long as you give appropriate credit to the original author(s) and the source, provide a link to the Creative Commons license, and indicate if changes were made.
In cases where reviewers are anonymous, credit should be given to 'Anonymous Referee' and the source.
The images or other third party material in this Peer Review File are included in the article's Creative Commons license, unless indicated otherwise in a credit line to the material. If material is not included in the article's Creative Commons license and your intended use is not permitted by statutory regulation or exceeds the permitted use, you will need to obtain permission directly from the copyright holder.
To view a copy of this license, visit https://creativecommons.org/licenses/by/4.0/

Point-by-point reply for NCOMMS-25-21445-T "TOFU-MAaPO: Fast, accurate and reproducible analysis of large metagenome sequence data from the Sequence Read Archive"

Reviewer #1 (Remarks to the Author):

There are six advancements that mark an inflection point in bioinformatics, and scientific computing more generally :

* Developing and maintaining code under distributed revision control
* Organizing software development with social programming systems
* Distributing software and its dependencies using packages managers
* Deploying software in encapsulated environments (i.e., containers)
* Coordinating execution with formal workflow languages
* Analyzing results in literate programming environments

These technologies are deeply interpenetrating, and when used together, produce multiplicative benefits to the scientific process. As a small example, the trivial effort that was required to download, install, deploy, run and examine the output of this software allowed this reviewer to quickly verify many of the key claims in this manuscript. This is not merely a technical advancement. It is a step forward for scientific inquiry.

```bash

 N E X T F L O W ~ version 24.10.5

Launching `https://github.com/ikmb/tofu-maapo` [distraught_mcnulty] DSL2 - revision: 36d8b0b796 [master]

=====================================================================
===============

 IKMB | TOFU-MAaPO | Version 1.5.0
```

===
===============

Nextflow Version: 24.10.5

Container Engine: singularity

=======INPUTS==
===============

Reads: : *_R{1,2}.fastq.gz

Host genome: : false

===
===============

Command Line: nextflow run ikmb/tofu-maapo -profile quickstart --reads '*_R{1,2}.fastq.gz' --cleanreads --outdir results

===
===============

No Host genome was specified!

[-] tofumaapo:QC:FASTQC_raw -

[-] tofumaapo:QC:TRIMREADS -

[-] tofumaapo:QC:CLEANREADS -

[-] tofumaapo:QC:COLLECTOR -

executor > local (3)

executor > local (5)

[4b/6cc43d] tof…o:QC:FASTQC_raw (PSM6XBR1) | 1 of 1 ✓

executor > local (7)

[4b/6cc43d] tof…o:QC:FASTQC_raw (PSM6XBR1) | 1 of 1 ✓

[90/ba325d] tof…po:QC:TRIMREADS (PSM6XBR1) | 1 of 1 ✓

[93/7c9eaf] tof…o:QC:CLEANREADS (PSM6XBR1) | 1 of 1 ✓

[aa/458b6e] tof…po:QC:COLLECTOR (PSM6XBR1) | 1 of 1 ✓

[5f/ad7445] tof…QC:FASTQC_clean (PSM6XBR1) | 1 of 1 ✓

[91/3d5245] tofumaapo:MULTIQC | 1 of 1 ✓

[10/9367f0] tof…aapo:SOFTWARE_VERSIONS (1) | 1 of 1 ✓

==

Duration: 13m 36s

==

Completed at: 21-Apr-2025 13:46:23

Duration : 13m 37s

CPU hours : 0.3

Succeeded : 7

```

We thank Reviewer #1 for executing and (successfully) checking our quickstart tutorial of TOFU-MAaPO on https://github.com/ikmb/TOFU-MAaPO, section "Quick start". The above command line output from Reviewer #1 confirms how easily TOFU-MAaPO can be installed and executed by a user without any bioinformatics expertise using a single command, namely "nextflow pull ikmb/tofu-maapo".

The main fault of this manuscript is that the authors *undersell* what they (and others) have achieved, and *understate* the implicit objectives of their own project. As the authors note, several other pipelines exist that, broadly speaking, do the same thing as TOFU-MAaPO. Normally, the introduction of yet another new tool into a crowded space would invite the XKCD 927 critique (https://xkcd.com/927/) : proliferating standards drives fragmentation of the community, thwarts comparative analysis and impedes forward momentum of scientific insight. The authors avoid this problem by making appropriate selections of tools, technologies and paradigms.

I would like to discourage the authors from viewing (and presenting) their software as Yet Another Analytical Pipeline for Metagenomes that competes in a field of similar alternatives. There are a finite number of reasonable analytical pathways from raw reads to annotated MAGs. There are a finite number of reasonable ways to structure those pathways and marshal execution of those tools. There are a finite number of reasonable ways to manage the input, intermediate and final data. Because the authors have elected to develop their software using a distributed revision control platform with integrated social programming features (Github), to use modular, encapsulated deployment environments (Singularity/Apptainer) and a formal workflow language (Nextflow), the result is that TOFU-MAaPO is not competing with ATLAS, MetaWRAP, nf-core/mag, or any of the dozens of other pipelines for metagenome analysis. It is in *conversation* with those projects.
```

Indeed, the authors based it on [an earlier workflow](https://github.com/marchoeppner/metagenomic-profiling), and it has already been forked at least five times. Concepts, implementations, and in many cases major chunks of code and subsystems can be (and often are) freely shared among these projects. There are not dozens of competing tools, but rather there is *one* community striving together to solve a common set of challenges. Tools like Github, Singularity and Nextflow make it practical for the community to have discussions about the merits of different approaches that are rooted in practice and grounded by data. TOFU-MAaPO is a statement within that conversation that welcomes further conversation, and this is a Very Good Thing. It is a mature thing. It is a responsible and healthy thing.

Of course, this is just my opinion. However, I suspect that the authors are very likely motivated by thoughts of a very similar nature. I would like to see those ideas centered a more clearly and stated more forcefully in the final version of this manuscript. As a community, I think it is long past time for us to come together and see what our combined efforts have yielded, and I wholeheartedly applaud the authors contribution to that effort.

We have implemented TOFU-MAaPO to be as modular and user-friendly as possible, enabling both software developers and end users to integrate, extend, or execute the pipeline with minimal effort. For example, installation requires no prior knowledge of microbiome analysis software. To emphasize this, we added to the Discussion, line 511: "TOFU-MAaPO is executable via single commands without the need to manually install and curate software packages. Each process downloads and executes only the required software container, allowing users to focus entirely on analyzing their metagenomic data."

Technical points

I have only one major criticism of the software itself. At the moment, TOFU-MAaPO can run either with input from SRA or with input from local FASTQ files, but not a combination of both. This is a very unfortunate limitation. In almost all cases, a researcher would want to place their data into context with relevant, previously published data. This is best practice, and should be a core feature.

I am not a Nextflow expert, but browsing the codebase does not suggest to me that it would be a major overhaul to make this possible.

We thank Reviewer #1 for pointing out that TOFU-MAaPO previously could not combine SRA and local FASTQ inputs. Thanks to the modular Nextflow implementation, this feature has now been added in version 1.5.1 (see https://github.com/ikmb/TOFU-MAaPO), allowing users to combine local and online FASTQ files, for example from the NCBI Sequence Read Archive (SRA), to be

combined in a single run. The relevant Methods section (line 227) and Figure 1 have been updated accordingly.

Line by line

77 : There may be countless ways to select and combine bioinformatics tools, but there are only a handful of *reasonable* ways in which they can be combined. Containers and workflows do not merely solve a technical problem, they solve a *scientific* problem by structuring discourse among human beings. Containers and workflows convert myriad decisions about inputs, outputs, configurations, versions, and parameters into a single object that can be discussed, criticized, applied, reused and improved upon. The fact that this leads to technical conveniences is a side effect!

We fully agree with this point. This is precisely why we have implemented our metagenome software pipeline in Nextflow and Singularity. Workflow languages and containers not only solve technical problems, but also structure scientific discourse by summarizing decisions about inputs, outputs, configurations, versions, and parameters into reproducible objects.

94-96 : Too may ideas are stuffed into a sentence that isn't up the task of holding them.

We have now split the sentence into several sentences to improve clarity: “For example, MetaWRAP [12] supports QC, taxonomic annotation, assembly, binning and bin refinement. Yet, it does not feature pathway annotation. The software implementation also lacks the integration of workflow management systems such as Nextflow or Snakemake with the resulting disadvantages for parallelization and error management.”

111 : A simple... what, exactly?

We apologize for the typo in this sentence. We have corrected the sentence as follows: “With minimal configuration based on Java, Nextflow and Singularity (quick installation also possible via conda), TOFU-MAaPO’s modules for quality control, taxonomical abundance, gene/pathway abundance and genome assembly can be launched with a single-command without the user having to manually install software environments or software packages (with the exception of downloading certain databases for specific tools).”

126-128 : Please discuss speed and accuracy in separate sentences. The parallel construction is confusing, and maybe a bit misleading depending on how you interpret it.

For clarity, we have separated both metrics (speed and accuracy) as follows: “TOFU-MAaPO produced 42% and 77% more high-quality bins (with score >=0.5) than those generated by the pipelines nf-core/mag and ATLAS with their bin refinement procedures, respectively. It also provided the fastest analysis among the tested pipelines.”

408 : Please correct the grammar "allows to create."

We have corrected the grammar of the following sentence: “TOFU-MAaPO allows users to create a list of different host genomes in a configuration file, enabling quick switching of host genomes between two runs.”

417 : Is "DAS tool" actually how you're supposed to say it? I'm not sure if it is supposed to be a German pun or not...

We thank Reviewer #1 for pointing out the name of the refinement tool. We have checked the original publication of the DAS Tool (https://doi.org/10.1038/s41564-018-0171-1) and adhered to the naming convention used there. For clarity, we now write “DAS Tool” instead of “DAS tool” and have updated this in the manuscript.

450-453 : No! Don't just assume that longer runtimes are are due to poorer parallelization! This is easily testable! If you want people to write better workflows, then please try to find out what the problems actually are!

We agree that attributing longer runtimes solely to poor parallelization is misleading. Our detailed analysis revealed additional contributing factors. In nf-core/mag (not discussed in the manuscript previously), the CONCOCT binning tool generates numerous library warnings, while unnecessary compression and decompression of FASTA files results in tens of thousands of processes, each requiring container provisioning. The latter resulted in 47,722 processes for compression alone in our benchmark for nf-core/mag. Thus, even if individual processes are fast, the overhead accumulates and prolongs runtime. TOFU-MAaPO addresses this by bundling multiple files or bins per container, processing them sequentially within a single container per sample and step. This balanced approach reduces overhead and ensures more efficient parallelization. We revised the manuscript (line 457) to clarify these distinctions and to emphasize that runtime differences result from both tool implementation and process management.

496 : Metagenomes are countable nouns, so they don't have amounts.

We revised the sentence to: "large numbers of metagenomes", reflecting precise scientific terminology and maintaining clarity.

501 : Docker? I thought it was Singularity/Apptainer? There's a docker image for the whole project, though. Is that what you're talking about here?

We appreciate the reviewer's attention to container details. TOFU-MAaPO defaults to Singularity/Apptainer for scientific usability, allowing execution without superuser privileges. Our implemented processing steps, e.g. MEGAHIT for assembly, use Docker containers compatible with Singularity/Apptainer. This approach ensures that users do not need to install Docker locally while benefiting from standardized containers. We clarified this distinction in the Methods (line 189) and Discussion (line 511), removing potential confusion regarding Docker usage.

506 : Typo. "quality or" should be "quality of."

Fixed

512 : This is not really true. TOFU-MAaPO sits at the beginning of the analytical cycle. Most of the important scientific questions lay downstream from MAGs, taxonomic annotations and functional annotations, and there are many other pipelines and tools the people would want to use after arriving at that point.

The original phrasing "explore" could mislead readers about TOFU-MAaPO's scope. TOFU-MAaPO primarily prepares metagenomic data into MAGs and annotated tables, providing a foundation for downstream analyses, statistical comparisons, and biological interpretations. We rephrased the sentence to emphasize the pipeline's role as a preparatory stage (line 524): "Users can process large metagenomic data from NCBI's SRA with a variety of tools implemented in a single pipeline. TOFU-MAaPO generates output tables and MAGs that serve as the basis for subsequent statistical and comparative analysis."

517-530 : This final paragraph is bit of a letdown. This software is super useful, and it is going to get more useful, and the things that will allow it to become more useful are exciting and important! The authors clearly understand this, but many of their intended users are focused on biological questions. Such folks may understand how the software works in a technical sense, but may not appreciate the impact these technologies are having on the research process in our field.

We revised the final paragraph (line 542) to better highlight biological relevance and future potential. TOFU-MAaPO enables direct creation of gene catalogs from downloaded metagenomes (e.g., via EggNOG integration in the future) and facilitates analyses previously possible only for large consortia. The paragraph now emphasizes how the pipeline opens opportunities for small research groups to conduct high-throughput metagenome analyses efficiently. We explicitly connect technological choices - Nextflow and Singularity - with the scientific impact, underlining usability, reproducibility, and scalability.

611 : Please fix the formatting of the equation in Figure 3. When using absolute value verticals, the parentheses are redundant. The LaTeX code should be something like $\log_{10}{\lvert \frac{\text{Bacteroides}}{\text{Prevotella}}\rvert}$.

We fixed the issue in the figure.

Final remarks

I've used some language in this review that could be taken to imply a major rewrite, but but want to be clear that I do not think that is the case. Almost everything amounts to asking the authors to more clearly articulate *why* they made these good decisions. I'd call that a minor revision.

We thank the reviewer for their suggestions on improving the clarity and functionality of our manuscript. We appreciate this feedback and have revised the manuscript accordingly to better explain technical details and the rationale behind our design choices. We believe that the revisions have substantially improved the readability and accessibility of the manuscript without changing its core content.

Reviewer #1 (Remarks on code availability):

This project is very close to what I would consider the Gold Standard in terms of reproducibility, documentation, availability and redistributability. It is actually rather difficult to imagine what more one could ask for without sounding churlish, but here's my wishlist :

[1] The inability to use mixed input from SRA and local files is unfortunate.

[2] More tests, maybe?

[3] More engagement on Github between the authors and other metagenomics workflows, maybe?

[4] More comments in the code

[5] Jupyter Notebook output

We sincerely thank the reviewer for considering TOFU-MAaPO as a near “Gold Standard” for reproducibility, documentation, availability, and redistributability. We greatly appreciate the constructive feedback and have addressed each point as follows:

[1] Mixed input from SRA and local files
We fully agree that the ability to combine local FASTQ files with online data from the NCBI Sequence Read Archive (SRA) is important for placing new data in context with previously published datasets. In response, we have now released TOFU-MAaPO version 1.5.1, which allows users to run a single workflow combining both local and online FASTQ input files. The Methods section (line 227) and Figure 1 have been updated to describe this functionality.

[2] Additional tests
Our manuscript includes both small-scale (100 metagenomes) and large-scale (17,090 metagenomes) examples, which demonstrate the reliability, scalability, and performance of TOFU-MAaPO. We believe these datasets sufficiently illustrate the robustness of the pipeline, while the modular design allows for easy extension with additional test cases in the future.

[3] Engagement on GitHub
To enhance visibility and foster community contributions, we plan to provide a web service version of TOFU-MAaPO, called TOFU-MAaPO-Web, hosted on our GDPR-compliant compute platform (https://hybridcomputing.ikmb.uni-kiel.de), which we intend to expand with additional metagenome tools. Then users without their own HPC resources can also perform the computations presented here.

[4] Code comment
We have added extensive comments to the Nextflow scripts to improve readability and ease of understanding for both Nextflow-experienced and new developers. This should make it simpler for users to adapt, extend, or integrate TOFU-MAaPO into larger workflows.

[5] Jupyter Notebook output
While we have not included Jupyter Notebook output for this version, the web service implementation described above will provide an accessible interface for running analyses without command-line expertise. Additionally, the modular design allows users to extract output tables and MAGs for downstream analysis, including visualization in Jupyter Notebooks if desired.

Reviewer #2 (Remarks to the Author):

This paper describes an automated workflow (TOFU-MAaPO) based on NetFlow and Singularity/Apptainer for metagenome analysis that encompasses automatic data download, read QC, assembly- and read-level analyses, with the former proceeding through to MAG quality assessment, taxonomic assignment and coverage estimation. In a benchmarking exercise against two comparable workflow products, ATLAS and nf-core/mag. The authors show that their software runs faster than either tested alternative work and imply that their workflow is superior due to the increased number, and quality profile, of the MAG catalogues that are recovered. The software can be installed and run (see below), and as such, the workflow is a useful contribution, particularly as it drops out both MAG and read level taxonomic- and functional-outputs and we would certainly consider using it in our laboratory for routine bulk processing of metagenome data.

We thank Reviewer #2 for their positive feedback and for their assessment that TOFU-MAaPO is a useful contribution for analyzing metagenome data.

Having said that, there are major concerns about the second claim made above, and the lack of balance in the presentation. The second claim referred to above, namely that TOFU-MAaPO "TOFU-MAaPO produced 42% to 77% higher quality MAGs" (line 38). While this is a statement of fact, in scientific terms I do not believe this claim has any meaning. It is simply a consequence of using five binning algorithms, rather than two or three, along with differences induced by different assemblers (SPADes vs MegaHIT), genome quality scoring (CheckM vs. CheckM2), dereplication and selection procedures (DasTools vs. MAGScoT) and MAG reporting criteria. This results from the fact that there are non-overlapping sets of MAG recovered by each method, and so, of course, you will see more MAGs if you incorporate more methods into an analysis (this can ascertained easily by making a presence-absence matrix of the non-redundant MAGs observed per workflow, using a suitable categorisation (e.g. secondary cluster in dRep). We do appreciate that the authors have taken effort to standardize the comparison as much as possible, but this is not a "benchmark" in any meaningful sense, because TOFU-MAaPO is inherently pre-biased to giving a better readout.

We agree that the number and type of binning algorithms affects MAG yield and quality. Our intention was not to imply that higher MAG counts are due solely to the number of tools, but to demonstrate the benefit of combining complementary binning approaches followed by a single, standardized refinement. TOFU-MAaPO integrates multiple binning outputs through MAGScoT, merging identical taxa bins and retaining only the most complete and least contaminated MAGs. This approach does not inflate counts through duplication but improves reconstruction of diverse taxa, particularly in complex samples.

To ensure comparability, we benchmarked only the **refined MAG sets** across all pipelines, not raw binning outputs. Quality assessment was performed exclusively with CheckM, a widely adopted standard in large-scale metagenomic studies (e.g., Almeida et al., Nat Biotechnol 2021, PMID 32690973; Zeng et al., Nat Commun 2022, PMID 36050292; Mayfach et al., Nature 2019, PMID 30867587). This allows objective comparison independent of internal pipeline structure. Our statement regarding the higher yield of high-quality MAGs refers specifically to this standardized comparison. We have clarified this in the manuscript (Introduction, line 129; Results, line 443).

On the technical systems level, this paper is presenting an overly optimistic picture of the utility of these tools in practice: specifically, their installation is often very system specific. For example, we tried to install and run TOFU-MAaPO on two HPC systems we use for our routine work on metagenomics; on one, the sys admin does not permit Singularity to be installed, even as a local installation. On the other, Singularity can be run, but external downloads are not permitted. We ended up running TOFU-MAaPO on a VirtualBox install of Linux on a Mac desktop machine, and it certainly worked on the example data, but in practical terms, we would not be able to use it on either of the servers for which we have routine access.

We appreciate the reviewer's effort to test installation. TOFU-MAaPO relies on standard containerization (Singularity), which enables reproducible execution across infrastructures without manual dependency installation. If Singularity or internet access is unavailable on a system, this limitation equally affects other pipelines such as nf-core/mag (requires Docker) and ATLAS (requires Conda) for their installation and execution. These are baseline requirements rather than unique obstacles of TOFU-MAaPO. On our GitHub page https://github.com/ikmb/TOFU-MAaPO, we have indicated that TOFU-MAaPO is compatible with any Linux system and requires only three basic dependencies: Java (for installation of Nextflow), Nextflow (the workflow manager) and Singularity (as container engine).

To avoid ambiguity, we have revised the Introduction (line 113) to clearly list the three minimal dependencies—Java, Nextflow, and Singularity—and note that installation is typically straightforward via Conda if needed: „With minimal configuration based on Java, Nextflow and Singularity (quick installation also possible via Conda), TOFU-MAaPO's modules for quality control, taxonomical abundance, gene/pathway abundance and genome assembly can be launched with a single-command without the user having to manually install software environments or software packages (with the exception of downloading certain databases for specific tools)."

The other disadvantage with these bundled workflows is that as time goes on, there is often a lot of work needed to locally maintain the installation and operation, due to

incompatibilities between new versions of the component software. The Discussion could have provided an informative and balanced view of these and related limitations, rather than simply highlighting the advantages.

We specifically addressed this by using Nextflow and containerized software. Nextflow enables single-command updates (nextflow pull ikmb/TOFU-MAaPO), while containers ensure stable execution despite evolving software dependencies. This design minimizes maintenance effort for users, which is now clarified in the Introduction. We modified the following sentence in the Introduction, line 80: „Nextflow and Snakemake increase scalability, portability, reproducibility and ease of use through the ability to process inputs/outputs of tasks as a data stream, support code management on sharing platforms such as GitHub, BitBucket and GitLab, support the development for workflow modules, sub-workflows, workflow compositions and automatic error handling, facilitate the continuation of an aborted or interrupted execution, provide visualizations of graph of software dependencies and executions, support the use of software containers such as Docker [7] and Singularity [8], and ensure portability, long-term software compatibility, and automated control of executions on high-performance compute clusters (HPCs).“

Reviewer #2 (Remarks on code availability):

The software was attempted to be installed on two HPC systems; one did not permit Singularity/Apptainer to the installed, while the other did not permit automatic downloaded. We tested the workflow (MAG analysis only) on Linux via VirtualBox installed on a Mac desktop machine, with confirmed operation.

As explained above, these issues reflect system-level restrictions, not the software. TOFU-MAaPO is compatible with any Linux system and only requires three minimal dependencies: Java (for installation of Nextflow), Nextflow, and Singularity. All other tools are provided in containers, ensuring reproducibility and ease of use across environments.

We thank Reviewer #2 for their careful evaluation and hope these clarifications demonstrate the fairness and technical robustness of our benchmarking, as well as the reproducibility and portability of TOFU-MAaPO.

Point-by-point reply for NCOMMS-25-21445A-Z "TOFU-MAaPO: Fast, scalable and reproducible analysis of large metagenome sequence data from the Sequence Read Archive"

Reviewer #1 (Remarks to the Author):

The authors have addressed all substantive points raised in my previous review. I am particularly pleased that they've implemented the ability to mix SRA records and local FASTQ files in an analysis.

We thank the reviewer for this positive assessment and are pleased that the implemented support for combining SRA records and local FASTQ files addresses this important point.

In light of the difficulties encountered by Reviewer 2, I tried running TOFU-MAaPO in a more restricted zone of our institute's computer cluster, where I did encounter a minor annoyance.

The issue is an unfortunate side effect of TOFU-MAaPO's single-command design, which aims to perform data retrieval and data processing concurrently controlled by the same command. This is, I think, the correct design philosophy, but unfortunately it won't always work smoothly in compute environments where compute resources and network access are partitioned by policy. Many (most?) HPC environments restrict public internet access from compute nodes. However, the software's error reporting and restartability allow users to work around the limitations of their compute environment if the workflow halts for this reason.

I think this is more of an documentation issue than a technical deficiency. I'm not a nextflow expert, but I think it is possible to use ``-entry`` or ``-stub-run`` to work within policy limitations fetch the data and pull the required containers. The user would marshal the data and containers on the head node of their HPC, and then dispatch the pipeline to compute nodes without public internet access. Some of the advantages of concurrent data retrieval and processing could be recovered by splitting the run into batches. Indeed, the authors describe this strategy in the manuscript!

We thank the reviewer for this thoughtful and constructive comment. We agree that this is primarily a documentation and deployment issue rather than a technical deficiency of TOFU-MAaPO itself. As a standard Nextflow workflow, TOFU-MAaPO is launched from the login/head node (that usually has internet access), while individual tasks are scheduled on the HPC by the local job scheduler. In restricted HPC environments with login/head nodes without internet access, internet-dependent steps may therefore require explicit routing or preparation depending on local policy.

To address this use case more explicitly, we have now added a dedicated documentation section on the GitHub page of TOFU-MAaPO entitled "Running on HPC Systems with restricted internet access". This section describes practical execution strategies for such environments, including setups in which container retrieval and internet-dependent download steps are performed separately from program executions, as well as configurations in which specific download jobs are routed to internet-enabled nodes or executed locally from the login/submission node. In our documentation, we also explain the newly added ``-stub-run``

feature, which allows users not only to test the pipeline in a dry run, but also to download containers, SRA input data, and databases without performing the full analysis. The documentation was added specifically to improve usability in such environments and to make these deployment strategies more transparent for users.

Ideally, users should not have to be Nextflow programmers to use this software. I encourage the authors anticipate and document common use cases that require the user to modify the workflow execution flow. For very common situations (like not having internet access on compute nodes), it's probably a good idea to wrap the needed Nextflow arguments in a custom argument, instead of relying on generic workflow semantics.

My only substantive request this time around is to explicitly address this use case in the documentation. Please give the section in the documentation a title that a frustrated user would recognize as a solution!

We agree that users should not need to know Nextflow to handle such scenarios, and the new documentation under “Running on HPC Systems with restricted internet access” has been added specifically to make these deployment strategies more transparent and to simplify their practical application in cases where internet restrictions exist on HPC systems.

(Very) minor points

There are a few turns of phrase in the manuscript that, while understandable, are a bit awkward. Some of these seem to come from applying Nextflow's rather peculiar use of specific words (e.g., "execution" and "implement") to general prose. There are also a few strange or incorrect constructions (e.g., "that allows to continue at a later date," line 164). I will list the ones I see, but it might be a good idea for the manuscript to get a brush-up by a copy editor before it goes to press.

We thank the reviewer for this helpful comment. We agree that some parts of the manuscript contained overly technical or awkward phrasing, and we have revised the text throughout for clarity and readability.

L. 63 : I don't quite understand what is meant by putting "clinical" is in parentheses. Phenotypes that may or may not be phenotypes of the host rather than the organism itself?

We agree that the use of "clinical" in parentheses was unclear in this context. Our intention was to refer to host- or disease-associated phenotype-specific microbial variation that may not be captured by broad reference genome catalogs. We have therefore rephrased the sentence as follows “… but naturally do not cover all ecological niches or host- and disease-associated microbal variation.”

L. 85, 87 : Grammatically, I think "executions" should probably be singular, but this is Nextflow's jargon for something like process, instance, task, or something like that. To me,

using "execution" as a noun always sounds like a reference to the death penalty. Please find a more generally used term.

We agree that the repeated use of “execution/executions” reflects workflow-system jargon and is awkward in general prose. We have therefore revised this sentence throughout to use more general and readable wording.

L. 99 : "lacks the use of" -> "does not use"

We fixed it.

L. 101 : "...assembly and binning based that fulfills..." Looks like some kind of copy editing error?

Fixed

L. 164 : "...that that allows to continue at a later point..." How about, "Nextflow workflows use automatic caching, and can be resumed after interruption from the last completed step. This feature can be leveraged feed output from previously completed runs into modules that were not included in the initial analysis, permitting users to perform additional analysis without starting the workflow from the beginning."

We thank the reviewer for this helpful suggestion. We agree that the original sentence was awkwardly phrased. We have revised it for clarity to better explain Nextflow’s caching and resume functionality in more general language.

L. 243 : Phix should be written phi X, Phi X or ΦX174.

Done

L. 325 : Implemented is not quite the right word (I think this is another bit of Nextflow jargon). I would say "packaged," "integrated," "adapted," or something like that.

Done

L. 388-391 : Suggested rephrase : For benchmarking, we used SRA project SRP102150, which has 100 SRA IDs. While TOFU-MAaPO can download and preprocess data from SRA itself (see Methods), both ATLAS and nf-core/mag required these IDs to be manually downloaded and staged. It was also necessary to generate a CSV file containing file path information for ng-core/mag.

Done

L. 403-404 : "...with a similar sample size led resulted in exceeding the available disk space..." -> "Because the default co-binning setting in the previous test exceeded our 3Tb limit for disk space, we elected to use single binning for ATLAS."

Done

L. 409 : "in order not to give TOFU-MAaPO an unfair speed advantage over ATLAS, which

also uses FastWC and BBDuk, although nf-core/mag uses the much faster fastp tool." I'm not sure why these confusing caveats are included. Just say which trimming method was used in each case, and mention separately what alternatives were available.

We have revised it to state more clearly which trimming method was used for each workflow, while moving the rationale and available alternatives to separate wording.

L. 442-444 : Rephrase in active voice.

Done

L. 459-465 : Thank you for pinpointing the root cause of the long run time in nf-core/mag. The phrasing is confusing, though. Is this what you mean? -> "The root cause of this surprisingly long run time is evidently due to the way the CONCOCT binning tool is applied within the nf-core/mag workflow, which results in a high overhead from spawning large numbers of utility processes (e.g., compression and decompression). The CONCOCT module in TOFU-MAaPO achieves substantially better performance (2.23 hours on with eight cores per sample verses 8.38 hours with on twelve cores per sample) by leveraging a more optimized parallelization strategy."

We agree that the original phrasing was confusing and have revised this passage for clarity. In the revised manuscript, we now describe more explicitly that the long runtime in nf-core/mag is largely attributable to the way the CONCOCT binning step is implemented there, together with additional overhead from many small utility processes

L. 470-473 : Proposed rephrase : ATLAS produced about half as many MAGs (XXX vs. YYY), using three fewer binning tools and a slightly longer running time.

We have revised it for clarity.

L. 504 : "without having to go through" -> "without the need to perform"

Done

L. 517 : Suggest "application of," "architecture," or "synthesis" rather than "implementation." The implementation belongs to the respective authors of those binning tools.

Done

L. 519-522 : The reason you get better results by integrating the results from five different binning methods is probably more subtle (and more interesting) than this. There are lots of papers on combining the results of multiple binners, and folks have been tinkering with this concept for at least 20 years. Just cite some of your favorites. Some recent papers :

- https://www.nature.com/articles/s41467-025-57957-6
- https://www.nature.com/articles/s41579-023-00981-4
- https://enviromicro-journals.onlinelibrary.wiley.com/doi/10.1111/1462-2920.16351

We thank the reviewer for this helpful suggestion. We agree that the original sentence was overly simplified. We have revised the Discussion to better reflect that the benefit of

combining multiple binners is a broader and more nuanced concept with prior literature, and we have added relevant references on benchmarking.

L. 531 : "...to keep up to date for processing and analyzing metagenomic data." The first half of this sentence is sufficient. You can delete this part.

We have shortened the sentence accordingly.

L. 534 : delete "still"

Done

L. 536 - 539 : Just say, "We are planning to add additional assembly tools and binning tools to TOFO-MAaPU."

Done

L. 543 : I don't mind the word "equip," but it's probably better to be consistent about what verb you use mean "adding a new piece of software to the workflow."

We have revised the sentence accordingly.

L. 545 - 549 : The fact that this workflow enables small research groups to perform analysis that previously required the labor of large consortia is a SUPER IMPORTANT POINT! I know this from years of personal experience working in the trenches of exactly these kinds of consortia. Better tooling isn't just a nice-to-have; better tooling enables better science. Please add it to the abstract!

We thank the reviewer for emphasizing this important point. We agree that one key contribution of TOFU-MAaPO is to make large-scale metagenomic analyses more accessible to individual research groups, including analyses that previously often required the coordinated effort of larger consortia. We have now added this point more explicitly to the Abstract and revised the Discussion accordingly.

To reflect this contribution more clearly throughout the manuscript, we have also revised the title to "TOFU-MAaPO: Fast, scalable and reproducible analysis of large metagenome sequence data from the Sequence Read Archive".

Reviewer #1 (Remarks on code availability):

My only substantive request is to add a short section in the documentation that explicitly address the use case where container pulls and data retrieval are performed separately. This is a use case that many HPC users will not realize they need until they encounter errors. Please give the section in the documentation a title that a frustrated user would recognize as a solution!

We thank the reviewer for this helpful and practical suggestion. We agree that this is an important use case for many HPC users and that it should be addressed explicitly in the

documentation. We have therefore added a dedicated section on the GitHub page of TOFU-MAaPO entitled “Running on HPC Systems with restricted internet access”, using a direct and solution-oriented title to make this guidance easy to find for users encountering such restrictions. The section describes how TOFU-MAaPO can be used in settings where container retrieval and internet-dependent data access must be configured separately from the main workflow execution (data processing).

Reviewer #3 (Remarks to the Author):

I have tested the code for TOFU-MAaPO, and I don't doubt the fluency of running it or its expected good performance and results. As commented by both reviewers, providing a good pipeline to integrate all the related tools is the major contribution of TOFU-MAaPO.

We thank the reviewer for testing TOFU-MAaPO and for acknowledging its smooth usability, expected performance, and value as an integrative pipeline for metagenomic analysis.

Regarding the comment about “TOFU-MAaPO produced 42% to 77% higher quality MAGs” from Reviewer #2, I agree with the reviewer. The performance gain is due to using more binning tools and different refinement tools, not due to the algorithmic innovation of TOFU-MAaPO itself. The statement that “TOFU-MAaPO produced 42% to 77% higher quality MAGs” is scientifically not fair to other pipelines, as they use different assembly, binning, and refinement tools. The authors should at least clarify that the performance gain is not a direct benefit of TOFU-MAaPO, but rather the result of combining different binning tools via MAGScoT -- and this weakens the contribution of TOFU-MAaPO.

We thank the reviewer for this important comment. We agree that the higher yield of high-quality MAGs in our benchmark should not be interpreted as the result of a novel standalone binning algorithm developed by TOFU-MAaPO itself. Rather, this result reflects the specific workflow configuration used in TOFU-MAaPO, namely the integration of multiple complementary binning tools followed by a unified refinement strategy using MAGScoT.

Our intention was not to attribute this gain to an inherent algorithmic innovation of TOFU-MAaPO as a single new binning method, but to demonstrate the practical benefit of a reproducible and modular workflow that combines complementary existing tools in a standardized manner. We agree that differences in assembler choice, binning composition, and refinement strategy contribute to the observed differences between workflows.

As noted in our response to Reviewer #1, we have revised the Discussion to better reflect that the benefit of combining multiple binners is a broader and more nuanced concept supported by prior literature, and we have added relevant references on metagenomic benchmarking.

To address this point explicitly, we have revised the manuscript to clarify this distinction and to avoid overstating the source of the performance gain. In particular, we changed the sentence in the Results section from “TOFU-MAaPO produced 42% and 77% more high-quality bins …” to “In our benchmark, the TOFU-MAaPO workflow yielded 12%, 42% and 77% more high-quality bins (when filtering with CheckM score ≥ 0.5) than metaFun, nf-

core/mag and ATLAS, respectively, after their respective bin refinement procedures, likely reflecting the combined effect of integrating multiple complementary binners with a unified refinement strategy."

We further revised the Abstract, Results, and Discussion to make clear that the observed benchmark advantage reflects the integration of multiple complementary binning tools with a unified refinement strategy within a reproducible workflow framework, rather than a novel standalone binning algorithm introduced by TOFU-MAaPO. We believe that this revised wording provides a more balanced and scientifically precise interpretation of the benchmark results.

In addition, contig binning and binning refinement are very competitive areas where "better" tools are constantly being developed. For example, based on our experience in metagenomics, COMEBin is currently the best contig binning tool for producing high-quality MAGs, better than all binning tools used in TOFU-MAaPO. Therefore, an established pipeline such as TOFU-MAaPO must be subject to frequent upgrades to modern tools in order to catch up with the state-of-the-art. This means the steps of the pipeline might be good today, but they could quickly become obsolete tomorrow. This is a limitation of all pipeline tools, so it's good that authors have discussed it in the Discussion.

We thank the reviewer for this important comment. We agree that contig binning and bin refinement are rapidly evolving areas and that metagenomic workflows should be able to incorporate improved methods over time. This consideration was a key motivation for the modular design of TOFU-MAaPO.

In response to the reviewer's suggestion, we have now incorporated COMEBin as an additional binning tool into TOFU-MAaPO and updated the benchmark analyses accordingly. Importantly, this extension could be implemented without major restructuring of the workflow, illustrating that TOFU-MAaPO can be readily adapted to methodological advances in the field.

We have revised the manuscript and Discussion to make this point more explicit. We believe this further strengthens TOFU-MAaPO as a flexible and extensible workflow framework rather than a static pipeline tied to a fixed set of tools.

The revised benchmark now includes the updated TOFU-MAaPO configuration with COMEBin, and the corresponding results are shown in revised Figure 2 and described in the Results section.

Regarding the comment about installing TOFU-MAaPO from Reviewer #2, it's worth mentioning that installing Singularity is painful, which was correctly complained about by Reviewer #2, though I don't think this is a critical fault of the software itself.
However, I must point out that the work of TOFU-MAaPO largely overlaps with a recent tool called metaFun (https://doi.org/10.1080/19490976.2025.2611544), which offers even more comprehensive analysis (such as strain-level analysis) and is well maintained and documented (https://metafun-doc.readthedocs.io/en/latest/index.html). metaFun also uses Nextflow but with Apptainer, avoiding the Singularity issues seen in TOFU-MAaPO by

Reviewer #2. The manuscript does not even mention metaFun, let alone benchmark against it. This is my major concern. This might be understandable as metaFun was published only 2 months ago, but it does weaken the contribution of TOFU-MAaPO.

We agree that container setup can represent a practical hurdle for some users, although this is not a critical limitation of TOFU-MAaPO itself. In the revised version, we have updated TOFU-MAaPO to use Apptainer and revised the GitHub documentation accordingly. Apptainer is the community-maintained successor of Singularity, so this update reflects an alignment with current community practice rather than a change in the underlying container-based workflow concept. (The pipeline can still be used with Singularity by enabling it in the configuration file.) We expect that this adjustment will further facilitate deployment in many Linux and HPC environments.

We would also like to clarify that the issue raised previously by Reviewer #2 did not reflect a software-specific limitation of TOFU-MAaPO itself. TOFU-MAaPO is executed as a standard Nextflow workflow from the login/head node (usually with internet access), whereas individual tasks are scheduled on the HPC by the local job scheduler. The reported issue therefore reflects cluster-specific deployment constraints, including local software installation policies and restrictions affecting container retrieval or data access during workflow execution on the headnode, rather than a limitation specific to TOFU-MAaPO.

To address this use case more explicitly, we have now added a dedicated documentation section on the GitHub page of TOFU-MAaPO named “Running on HPC Systems with restricted internet access” describing how the workflow can be deployed in HPC environments with restricted internet access, including execution strategies in which container retrieval and data processing are prepared separately. This documentation was added specifically to improve usability in such environments and to make these deployment strategies more transparent for users.

We further thank the reviewer for pointing out the recently published metaFun workflow. Because metaFun appeared only after the initial submission of our manuscript, it was not discussed in the original version. We have now cited and discussed metaFun in the revised manuscript and have extended the benchmark accordingly. This allows a more complete and up-to-date comparison with current Nextflow-based metagenomic workflow frameworks.

We believe these revisions address the reviewer’s major concern and strengthen the manuscript substantially.

Therefore, while I do appreciate the authors' efforts to put these metagenomic analysis tools together into a good pipeline tool, the contribution is not sufficient for publication in Nature Communications. I would only recommend publication if the 1,652 non-redundant species-level genome bins of IBD mentioned in the Introduction were constructed using TOFU-MAaPO and published with this paper, rather than in previous work.

We thank the reviewer for this comment. We agree that constructing and publishing a large new MAG resource would represent a different type of contribution. However, the main aim of the present manuscript is to describe and benchmark TOFU-MAaPO as a reproducible, modular, and scalable workflow for large-scale metagenomic analysis, rather than to present a new biological resource manuscript centered on a newly generated MAG catalog.

One key contribution of TOFU-MAaPO is to make large-scale metagenomic analyses more accessible to individual research groups, including analyses that previously often required the coordinated effort of larger consortia. We have now added this point more explicitly to the Abstract and revised the Discussion accordingly.

The previously published set of 1,652 non-redundant species-level genome bins from IBD-associated metagenomes is cited in the Introduction as an example of the type and scale of analyses that workflows such as TOFU-MAaPO are intended to support, but it is not the central contribution of the present study. In addition, the underlying IBD cohort data are not an openly accessible benchmark dataset comparable to the SRA-based datasets used here and were therefore not intended as the basis for the workflow benchmark presented in this manuscript. We have revised the Introduction to make this distinction clearer.

We have also further strengthened the methodological scope of the manuscript through the updated benchmark, integration of COMEBin, transition to Apptainer, and inclusion of metaFun in the comparison and discussion.
